# Detection of endoplasmic reticulum stress and the unfolded protein response in naturally-occurring endocrinopathic equine laminitis

**DOI:** 10.1186/s12917-018-1748-x

**Published:** 2019-01-10

**Authors:** Lynne Cassimeris, Julie B. Engiles, Hannah Galantino-Homer

**Affiliations:** 1 Department of Biological Sciences, Lehigh University, Bethlehem, PA 18015 USA; 2 Department of Clinical Studies/New Bolton Center, School of Veterinary Medicine, University of Pennsylvania, Kennett Square, PA 19348 USA; 3 Department of Pathobiology/New Bolton Center, School of Veterinary Medicine, University of Pennsylvania, Philadelphia, PA 19104 USA

**Keywords:** Horse, Lamella, Hoof, Insulin, Cell stress

## Abstract

**Background:**

Laminitis is often associated with endocrinopathies that cause hyperinsulinemia and is also induced experimentally by hyperinsulinemia, suggesting that insulin initiates laminitis pathogenesis. Hyperinsulinemia is expected to activate pro-growth and anabolic signaling pathways. We hypothesize that chronic over-stimulation of these pathways in lamellar tissue results in endoplasmic reticulum stress, contributing to tissue pathology, as it does in human metabolic diseases. We tested this hypothesis by asking whether lamellar tissue from horses with naturally-occurring endocrinopathic laminitis showed expression of protein markers of endoplasmic reticulum stress.

**Results:**

Three markers of endoplasmic reticulum stress, spliced XBP1, Grp78/BiP and Grp94, were upregulated 2.5–9.5 fold in lamellar tissues of moderately to severely laminitic front limbs (*n* = 12) compared to levels in controls (*n* = 6–7) measured by immunoblotting and densitometry. Comparing expression levels between laminitic front limbs and less affected hind limbs from the same horses (paired samples from 7 to 8 individual horses) demonstrated significantly higher expression for both spliced XBP1 and Grp78/BiP in the laminitic front limbs, and a similar trend for Grp94. Expression levels of the 3 markers were minimal in all samples of the control (*n* = 6–7) or hind limb groups (*n* = 7–8). Immunofluorescent localizations were used to identify cell types expressing high levels of Grp78/BiP, as an indicator of endoplasmic reticulum stress. Grp78/BiP expression was highly elevated in suprabasal epidermal keratinocytes and only observed in laminitic front limbs (10/12 laminitic samples, compared to 0/7 in sections from the hind limbs and 0/5 of controls).

**Conclusions:**

These data demonstrate that the endoplasmic reticulum stress pathway is active in naturally occurring cases of laminitis and is most active within a subset of epidermal keratinocytes. These data provide the rationale for further study of endoplasmic reticulum stress in experimental models of laminitis and the links between laminitis and human diseases sharing activation of this stress pathway. Pharmacological options to manipulate the endoplasmic reticulum stress pathway under investigation for human disease could be applicable to laminitis treatment and prevention should this pathway prove to be a driver of disease progression.

**Electronic supplementary material:**

The online version of this article (10.1186/s12917-018-1748-x) contains supplementary material, which is available to authorized users.

## Background

Equine laminitis is a common, painful, and debilitating disease and a significant cause of mortality in horses [1]. The hallmark of laminitis has traditionally been described as the loss of attachment between the epidermal lamellae of the inner hoof wall and the interdigitating dermal lamellae of the underlying distal phalanx [2]. However, it has become increasingly apparent that laminitis is an extremely complex disease that includes lesions of multiple tissues of the foot, that these lesions and inflammation vary with underlying cause and disease duration, and that all cases have failure of the suspensory apparatus of the distal phalanx as a common feature [3–6]. Concurrent with this failure, the bone, dermal and epidermal tissues of the foot are stretched, distorted, and crushed, resulting in abnormal hoof growth and secondary pathologies such as foot abscesses, bone loss and fractures of the distal phalanx [4], significant epidermal lamellar pathology that is apparent histologically at both acute and chronic stages of laminitis [4, 5, 7] and that is grossly evident at chronic stages as the lamellar wedge lesion [8], and neuropathic pain [9].

Several classes of extrinsic or intrinsic factors can trigger laminitis, including prolonged load due to a painful, and therefore non-weight-bearing contralateral limb injury (supporting limb laminitis; [10]), toxicosis [11]; sepsis [12, 13], or endocrinopathies, including equine metabolic syndrome (EMS), commonly associated with obesity, regional adiposity, and laminitis [14–16] and pituitary pars intermedia dysfunction (PPID), also termed equine Cushing’s disease [17]. The latter endocrine-based triggers are associated with hyperinsulinemia, reflecting a failure to properly regulate insulin levels in response to carbohydrate challenge, and represent the most common cause of laminitis [18, 19]. Moreover, an experimental euglycemic-hyperinsulinemic clamp protocol has been shown to induce laminitis in horses and ponies [20, 21]. Recent studies have indicated that hyperinsulinemia is the driving force underlying endocrinopathic laminitis (EL) [22–24]. Although the lamellar microanatomy is not found in humans, we expect that laminitis shares cell-based disease mechanisms with one or more human diseases. Given that the insulin dysfunction in horses with EMS and a subset of the PPID cases resembles human metabolic syndrome and type 2 diabetes in many ways, and the growing realization that endoplasmic reticulum (ER) stress and the resulting unfolded protein response (UPR) play critical roles in loss of cell function in diabetes and associated diseases [25–30], we hypothesized that ER stress and UPR contribute to EL pathogenesis.

ER stress and the UPR have been identified as critical cellular stress response pathways in a number of human diseases and are a topic of intense scrutiny for therapeutic interventions. In addition to type 2 diabetes, ER stress/UPR have been implicated in neurodegenerative disorders, type 1 diabetes, neuropathic pain, cancer and skin diseases [25, 26, 29–36]. ER stress and the UPR result when the synthesis of new membrane and/or secreted proteins overwhelms the protein folding capacity within the ER lumen. Misfolded proteins are detected by three sensors in the ER membrane and these relay signals to the cytoplasm and nucleus, which result in increased synthesis of ER chaperones and other proteins required for protein folding in the ER. Prolonged ER stress and UPR can lead to apoptosis if cell homeostasis is not restored [27, 30, 32]. The primary objective of this study was to evaluate expression of 3 ER stress markers in lamellar tissue from horses with naturally-occurring EL in front limbs relative to the front limbs from non-laminitic controls using commercial antibodies that cross react with the homologous equine proteins for immunoblotting and immunohistochemical techniques. An additional objective was to compare expression of the same 3 markers between EL affected front limbs and the less affected hind limbs from the same horses, to distinguish between systemic changes common to all limbs and changes associated specifically with moderate to severe laminitis. We hypothesized that the expression of these markers is elevated in the EL front feet due to the activation of the ER stress pathway and the UPR, compared to either control front feet or the less affected hind feet from the EL cases. The ER stress markers investigated were: the spliced form of X-box binding protein 1 (XBP1s), a transcription factor that activates expression of genes for ER chaperones [29, 30, 37] and two ER chaperones known to be upregulated in ER stress/UPR, 78 kD Glucose-regulated Protein (Grp78, also known as Binding immunoglobulin protein (BiP)) and 94 kD Glucose-regulated protein (Grp94) [38, 39]. Grp78/BiP was also used to localize specific cell type(s) in lamellar tissue undergoing ER stress.

## Results

### Overview of the experimental groups

A cohort of naturally-occurring EL cases [40] was used for detection of potential ER stress/UPR markers. Of the twelve EL cases tested here, 6 were diagnosed with OB/RA, which, along with insulin dysregulation and laminitis, are clinical and historical features of EMS in horses and ponies [18, 41–43], and 6 with PPID (Table 1). In the EL cases, front limbs were most severely affected, all having gross as well as both quantitative and qualitative histological lesions compatible with moderate to severe chronic-active laminitis, and were designated as “EL Front.” In contrast, the hind limbs from EL cases (designated “EL Hind”) either had no lesions compatible with laminitis (six limbs) or had lesions only detectable by qualitative histology (four limbs), compatible with mild/sub-clinical laminitis, and thus were comparable to control feet, as detailed in Table 1 and in (see Additional files 1, 2, 3, 4, 5, 6, 7 and 8). Of the age-matched controls, none had clinical signs, gross lesions, or quantitative histology lesions consistent with laminitis, but two feet in the group did have qualitative microscopic lesions compatible with mild or subclinical laminitis (Tables 1, 2; see Additional files 2, 3, 4, 5, 6, 7 and 8). Given the subtlety of these lesions, characterized by focal or multifocal disruption of qualitative, not quantitative architectural parameters, and that, in the authors’ experience, and in other reports of naturally occurring laminitis, these lesions are commonly encountered in feet considered “normal,” particularly in aged horses [4, 22], these lesions were considered incidental, and the feet were not excluded as controls. As detailed in (see Additional files 1, 2, 3, 4, 5, 6, 7 and 8), the following pathological features were significantly increased in the EL front group in comparison to the Control and EL hind groups: Gross Pathology Severity Score, Stratum Internum-Corium Measurement (SICM) at all locations, PEL length, KA displacement, SDL displacement at all locations, and Overall (additive) Qualitative Histopathology Score. Of the specific qualitative histopathology lesions evaluated in generating the overall score, EL front limbs had increased distribution scores, relative to control feet, for the following: lamellar epidermal basal cell nuclear polymorphism, basement membrane lesions, displacement of KA relative to SELs, SEL morphology, epidermal lesions including hyperplasia, metaplasia, and orthokeratosis, dermal spindle cell hypertrophy, white blood cell perivascular infiltration (with increased detection and distribution scores for lymphocytes, histiocytes, plasma cells, hemosiderophages, and polymorphonuclear leukocytes), and vascular endothelial hypertrophy and abaxial apoptotic/necrotic cells. Others have reported the presence of leukocytes in lamellar tissue from the euglycemic-hyperinsulinemic clamp experimental model of laminitis, albeit fewer than in sepsis models [7, 44], and, to a lesser extent, in natural EL cases [7, 22]. However, neither these previous studies nor this study provides quantitative data using specific differentiation markers to allow comparison. In the naturally occurring disease, inflammatory cells may reflect secondary lesions of laminitis (solar prolapse, toe abscesses) or a response to dermal and epidermal lamellar tissue damage and cellular activation in these moderately-to-severely affected feet, and perhaps these lesions were less prevalent in the earlier studies. Similar to previous reports [7, 22], the EL front foot distribution scores for basement membrane lesions and Mitotic Figure score for the axial region of the PEL were significantly increased relative to control, but not to EL hind limbs, perhaps suggesting that these lesions are early or sub-clinical histopathological lesions of EL.

**Table 1:** Sample data summary of signalment, laminitis risk factor, pathology category, and ER stress/UPR marker data

Samples (case #, limb)	Age (yr)	Breed	Sex	Laminitis Risk Factor	Laminitis Category	Immunoblot Relative Band Intensities (arbitrary units**)**			Grp78/BiP Localization
						XBP1s	Grp78/BiP	Grp94	
Control									
61 RF	11	WB	MC	NL/O	SUB	26.8	15.7	12	nd
92 LF	18	WB	F	NL/O	SUB	24.7	11.4	17.1	nd
102 LF	17	QH	F	NL/O	NL	38.4	0.9	25	negative
110 LF	14	TB	MC	NL/O	NL	9.7	nd	17.2	nd
111 LF	18	TB	F	NL/O	NL	nd	nd	nd	negative
113 LF	12	TB	F	NL/O	NL	27.6	0.5	17.2	negative
114 LF	12	TB	F	NL/O	NL	5.2	2.9	26.5	negative
129 RF	22	AR	F	NL/IF	NL	5.8	18.4	42.8	negative
Mean ± sd						19.7 ± 12.9	8.3 ± 7.9	20.8 ± 10.7	
EL Front Limb									
63 RF	33	MO	F	PPID	SEV	79.1	12.8	17.4	nd
63 LF	33	MO	F	PPID	SEV	nd	nd	nd	AbAx
73 LF	6	TBX	F	OB/RA	MOD	36.6	20.5	13.2	negative
75 RF	22	MO	F	PPID	SEV	96.5	250	106	nd
75 LF	22	MO	F	PPID	SEV	nd	nd	nd	AbAx, Mid (KA)
90 LF	21	MH	M	OB/RA	MOD	54.3	1.9	41.5	nd
101 RF	13	MO	MC	OB/RA	SEV	16	79	91.5	AbAx, Mid (KA)
104 RF	23	WB	MC	PPID	SEV	36.6	1.3	24.5	AbAx, Mid (KA)
109 LF	9	QH	F	OB/RA	SEV	19	23.6	27.9	AbAx
116 LF	8	QH	F	OB/RA	SEV	100	131.2	100	nd
116 RF	8	QH	F	OB/RA	SEV	nd	nd	nd	AbAx, Mid (KA)
134 RF	18	TB	MC	PPID	SEV	87.3	100	57.9	negative
134 LF	18	TB	MC	PPID	SEV	nd	nd	nd	AbAx, Mid (KA)
140 LF	14	TBX	F	OB/RA	SEV	12	15.1	36.9	AbAx, Mid (KA)
141 LF	21	RMH	F	PPID	SEV	72.6	296	70.1	AbAx, Mid (KA)
141 RF	21	RMH	F	PPID	SEV	nd	nd	nd	AbAx, Mid (KA)
165 LF	23	WB	F	PPID	MOD	23	15	31.6	nd
Mean ± sd						52.8 ± 33.0	78.9 ± 100.2	51.5 ± 32.9	
EL Hind Limb									
63 LH	33	MO	F	PPID	SUB	nd	nd	nd	negative
73 LH	6	TBX	F	OB/RA	NL	19.4	15	39.7	negative
75 RH	22	MO	F	PPID	NL	17.8	1.7	33	negative
101 LH	13	MO	MC	OB/RA	SUB	nd	nd	nd	negative
104 RH	23	WB	MC	PPID	SUB	7.7	7.6	21.1	nd
109 RH	9	QH	F	OB/RA	NL	nd	4.7	27	negative
116 RH	8	QH	F	OB/RA	SUB	1.8	5.2	17.4	negative
134 RH	18	TB	MC	PPID	NL	3.8	33	25.4	negative
141 RH	21	RMH	F	PPID	NL	0.1	5.5	17.7	nd
165 LH	23	WB	F	PPID	NL	9.4	5.4	23.8	nd
Mean ± sd						8.6 ± 7.6	9.8 ± 10.2	25.7 ± 7.6	

legend: Summary of EL cases and controls used in the present study. Case numbers were assigned at the time of tissue collection, with foot identified as left front (LF), right front (RF), left hind (LH), or right hind (RH). Age listed in years. Breeds include: American Quarter Horse (QH); Arabian (AR); Miniature Horse (MH); Morgan (MO); Rocky Mountain Horse (RMH); Thoroughbred (TB); Thoroughbred cross (TBX; case 73 is TB/Clydesdale cross; case 140 is TB/WB cross); Warmblood (WB). Sex is listed as female (F), male (M), or male castrate (MC). Diagnosis includes non-laminitic, other orthopedic issues (NL/O); non-laminitic, infertility (NL/IF); PPID; obesity/regional adiposity (OB/RA). Laminitis categories include non-laminitic (NL); sub-clinical (SUB), Moderate chronic-active (MOD), and Severe chronic-active (SEV) based on the criteria presented in Table 2 and Methods. Relative immunoblot band intensities were normalized and calculated as described in methods. The mean ± standard deviation (sd) is given for each marker. EL Front Limbs differed from Controls (*p* = 0.007 (XBP1s), 0.03 (Grp78/BiP), 0.009 Grp94) and from EL Hind Limbs (*p* = 0.006 (XBP1s), 0.05 (Grp78/BiP). Anti-Grp78/BiP labeled sections were scored visually for detectable expression. Positive antibody labeling is noted by location along the PEL axis: abaxial (AbAx), middle (Mid), keratinized axis (KA). nd, not determined

**Table 2:** Definition of laminitis categories based on the duration of clinical signs, gross pathology, and histopathology

Pathology Category	Duration (days)	Gross Score	Laminitis Histopathology
Non-Laminitic	0	1	–
Sub-Clinical	0	1	+
Moderate	> 7	2 or 3	+
Severe	> 7	4	+

legend: Laminitis pathology categories for limbs from cases and control horses, based on the duration of clinical signs of laminitis in days, gross pathology severity score (1–4), and presence or absence of histological lesions consistent with laminitis, as described in detail in Methods. See Table A1 (see Additional file 1) for duration and gross score, and Tables A4-A8 (see Additional files 4, 5, 6, 7 and 8) for qualitative scoring used to determine presence or absence of histological lesions consistent with laminitis for each limb

### Immunoblotting to detect expression of ER stress/UPR markers

**Fig. 1:**
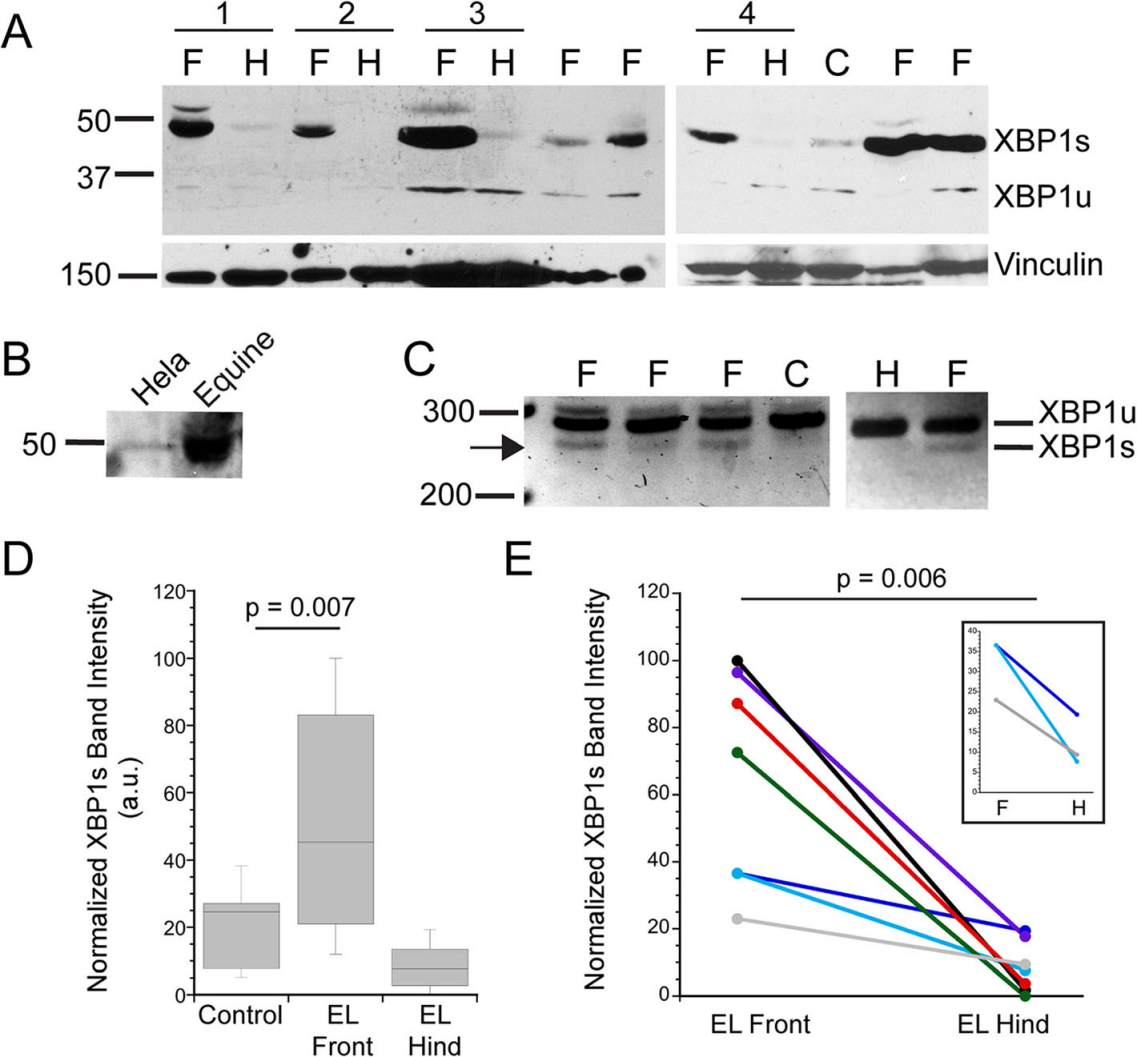
Spliced XBP1 is expressed in laminitic tissues. **a**. Representative immunoblots probed for expression of the XBP1s or XBP1u proteins derived from spliced and unspliced XBP1 mRNA, respectively. XBP1s is prevalent in EL front limbs (F) and barely detectable in either EL hind limbs (H) or controls (C). Membranes were reprobed for vinculin as a loading control. Four pairs of EL front and hind limbs from the same horses are noted by horizontal bars and numbered 1–4 (pair 1: 116 LF, RH; pair 2: 141 LF, RH; pair 3: 134 RF, RH; pair 4: 104 RF, 104 RH; see Table 1). **b**. The antibody to XBP1 recognized a band of the same mw in both Hela (human) cells and equine lamellar tissue. **c** PCR amplification of a region near the XBP1 splice site produces amplimers of the expected base pairs for spliced and unspliced XBP1. Samples are labeled as in (**a**). **d**,**e**. Immunoblot band intensities for XBP1s were measured and normalized as described in Methods. **d** Box blot summarizing XBP1s expression levels in control (*n* = 7), EL front (*n* = 12) and EL hind limbs (*n* = 7). Horizontal lines represent the median value for each group. XBP1s expression was not significantly different between control and EL hind limbs (*p* = 0.08). **e** Comparison of XBP1s expression levels in paired samples from EL front and EL hind limbs of the same horses (*n* = 7 pairs), inset shows the 3 sample pairs with similar XBP1s band intensities between front (F) and hind limb (H). Although the differences between front and hind limbs are smaller, the same pattern is apparent as that in pairs having much greater variation

The first marker of ER stress/UPR that we examined was XBP1s, a transcription factor expressed when its mRNA is spliced in the cytoplasm by inositol requiring enzyme 1 (IRE-1), an ER membrane protein active as a splicing factor in response to ER stress [28–30]. Splicing removes 26 nucleotides from the XBP1 message, changing the frame of the transcript and resulting in expression of an ~ 50 kD protein [37]. Unspliced XBP1 mRNA is expressed at low levels and encodes a protein of ~ 30 kD. The presence of the 50 kD XBP1s is often used as a marker of ER stress/UPR [27, 29, 37, 45]. As shown in Fig. 1a, XBP1s is expressed at high levels in tissues from many EL front limb samples compared to that in samples from control front limbs or the EL hind limbs. Importantly, matched EL front and hind limbs from the same individual horses showed robust expression of XBP1s in the EL front limb sample, but only minimally detected this protein in the EL hind limb (Fig. 1a). We confirmed the identity of the 50 kD immunoreactive band by demonstrating that the equine protein co-migrated with the human protein (protein lysate from Hela cell line; Fig. 1b). In addition, PCR primers were designed to flank the spliced region of the mRNA and amplify sequences of 281 base pairs (unspliced) and 255 base pairs (spliced). As shown in Fig. 1c, amplimers of both spliced and unspliced XBP1 cDNAs were detected in laminitic samples. The PCR results are not quantitative and served only to confirm the immunoblot results.

To allow a more quantitative comparison across a number of separate immunoblots, we normalized band intensities as described in Methods. We confirmed that the normalization procedure yielded reproducible values by plotting normalized XBP1s band intensities from samples run on two separate occasions (see Additional file 9: Figure S1A and Additional file 10: Legend to Figure S1A). As shown in Fig. 1d, XBP1s levels were higher in lamellar tissue from EL front limbs (*n* = 12) compared to levels in the control front limbs (*n* = 7). For the EL front limbs, similar levels of XBP1s were detected for either PPID or obesity/regional adiposity laminitis risk factors (*p* = 0.181, data summarized in Table 1). In contrast to the difference between EL and control front limbs, XBP1s levels in control and EL hind limbs were not significantly different (Fig. 1d). We also compared XBP1s band intensity in paired samples from the same horses. XBP1s expression was again higher in EL front limbs compared to a corresponding hind limb (n = 7 paired samples) (Fig. 1e). The inset in Fig. 1e expands the y-axis for three EL cases that had similar XBP1s expression in the front and hind limbs to show that the pattern of higher expression in the front limb relative to the hind limb is still observed. For either comparison group, the EL front limbs showed greater variation in XBP1s expression levels compared to that in either controls or the EL hind limbs (Table 1).

**Fig. 2:**
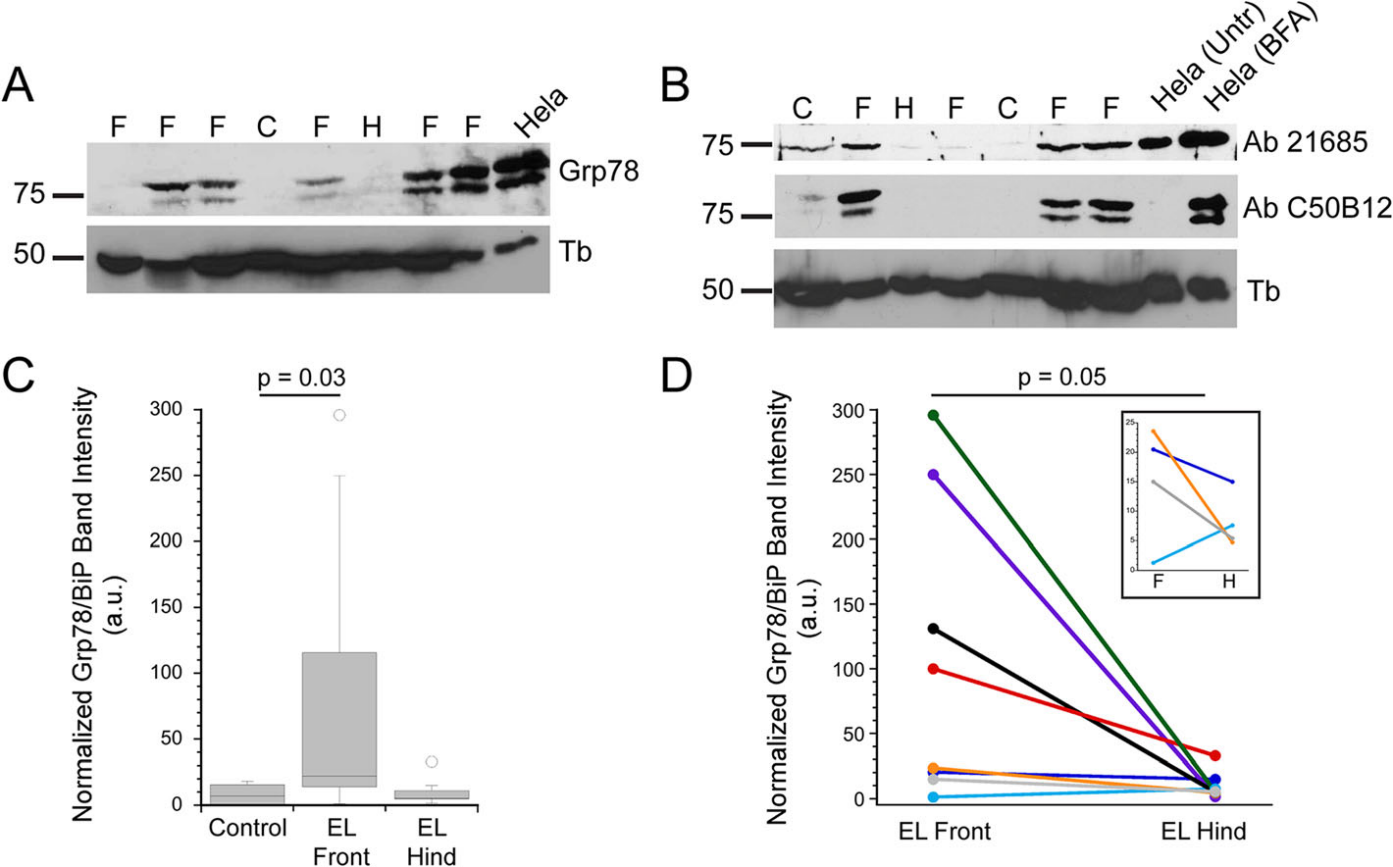
Grp78/BiP is expressed at higher levels in laminitic tissues. **a**. Representative Grp78/BiP immunoblot, reprobed for alpha-tubulin as a loading control. A band of ~ 78 kd co-migrates with the Hela (human) protein. **b**). Two commercial antibodies to Grp78/BiP yield qualitatively similar results. Each antibody detects upregulation of Grp78/BiP in Hela cells (human cell line) treated with brefeldin A (BFA) to induce ER stress (see Methods and (Additional file 9: Figure S1 and Additional file 10: Legend to Figure S1A)). Lanes in (A,B) are labeled as: control (C), EL front limb (F) and EL hind limb (H). (**c**). Box plot of normalized Grp78/BiP band intensities in control (*n* = 6), EL front limbs (=12) and EL hind limbs (*n* = 8). Horizontal lines represent the median value for each group. Grp78/BiP expression was not significantly different between controls and EL hind limbs (*p* = 0.77) **d**. Comparison of Grp78/BiP expression in paired samples (*n* = 8) from EL front and hind limbs from the same horses. The inset shows the 4 sample pairs with small differences in Grp78/BiP band intensities between EL front and hind limb samples. The same pattern is apparent in 3 of 4 pairs, where the value for Grp78/BiP level in the front limb is greater than that in the hind limb

Since ER chaperones are also upregulated during ER stress/UPR [26, 29, 30, 32], we next examined Grp78/BiP expression levels. The level of Grp78/BiP was elevated in many EL front limbs, while only weakly detected in controls (Fig. 2a-c). Two commercial antibodies gave qualitatively similar results, and each antibody recognized a human protein of the same size as the equine protein, indicating that the antibody is recognizing the same protein in human and equine tissues (Fig. 2a, b and (see Additional file 9: Figure S1B and Additional file 10: Legend to Figure S1A)). In some cases we detected a protein doublet, rather than a single band, but do not know whether this reflects a post-translational modification to Grp78/BiP. The doublet was also detected in samples from a human cell line (Hela; Fig. 2a, b). Comparing immunoblot band intensities between control (*n* = 6) and EL front limbs (*n* = 12) demonstrated higher expression in laminitic samples (Fig. 2c, Table 1), while controls and EL hind limbs (*n* = 8) did not differ significantly in Grp78/BiP expression levels. Comparison of Grp78/BiP expression between paired tissue samples from EL front and hind limbs (*n* = 8 horses) also demonstrated higher expression in the laminitic front limbs compared to the hind limbs (Fig. 2d). The pattern of higher expression in the EL front limbs was robust for 4 of 8 samples, with the same pattern observed for 3 of the remaining 4 sample pairs (inset Fig. 2d). The Grp78/BiP levels in EL front limbs from PPID or obesity/regional adiposity risk factors were not significantly different (*p* = 0.278; data summarized in Table 1). As observed for XBP1s expression, the Grp78/BiP levels showed a much broader distribution in laminitic samples compared to the levels in controls or the EL hind limbs (Fig. 2c, d).

**Fig. 3:**
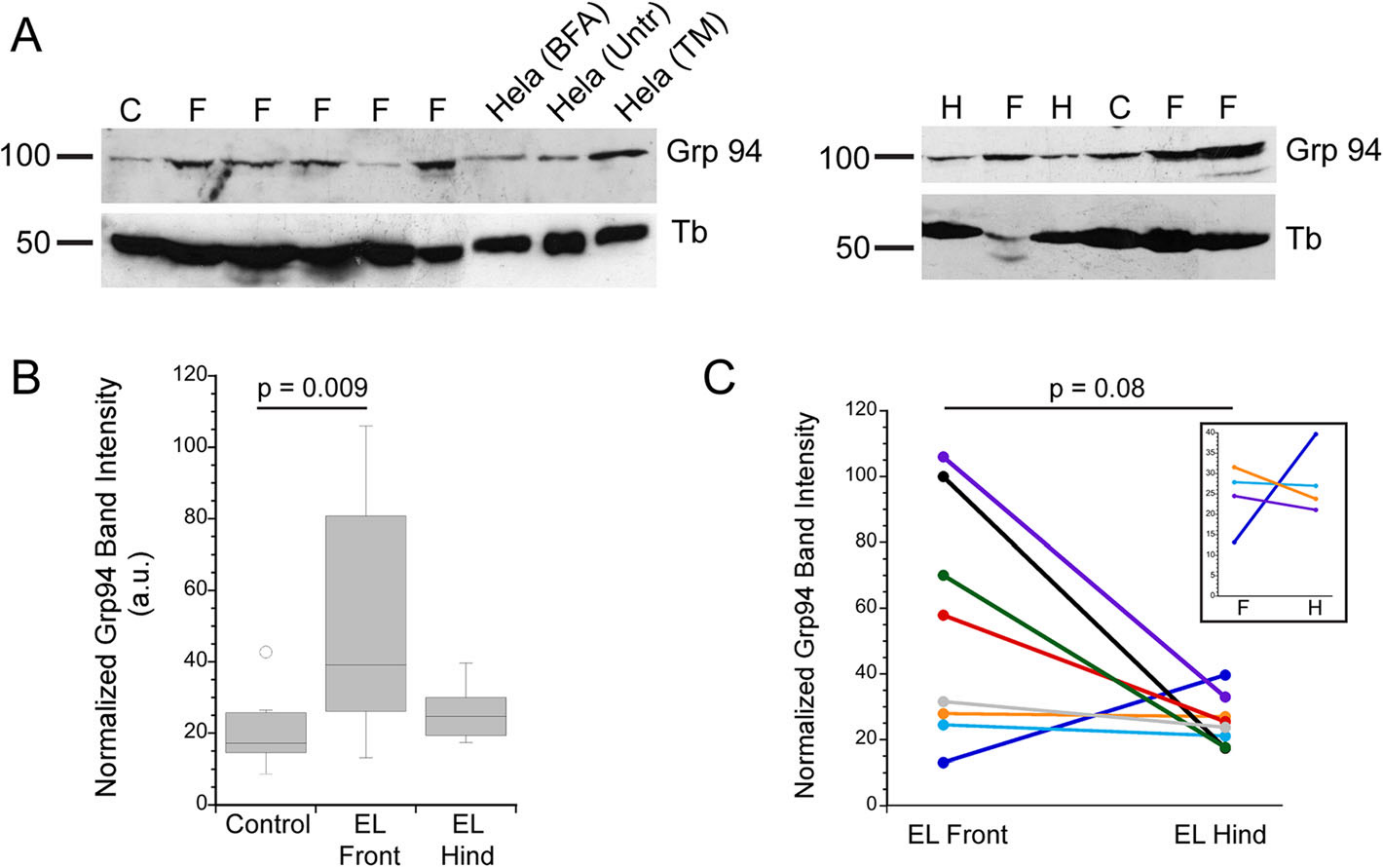
Grp94 is expressed at higher levels in many laminitic samples. **a**. Representative immunoblots of lamellar tissue from control (C), EL front limbs (F) and EL hind (H) limbs. The anti-Grp94 antibody recognizes a band of the same mw in Hela (human) cells, and this band is upregulated by treatment with tunicamycin, an inducer of ER stress (see Methods). **b**. Box plot of normalized Grp94 immunoblot band intensities showing higher expression in laminitic EL front limbs. Horizontal lines represent the median value for each group. **c** Comparison of Grp94 expression in paired EL front and hind limbs from the same horses (*n* = 8). The inset shows an expanded region of the Y axis

Immunoblots of Grp94, another ER chaperone [32, 39], were used to further test whether laminitic tissues express markers of ER stress/UPR. Higher levels of Grp94 were detected in EL front limbs (*n* = 12) compared to the lower level present in controls (*n* = 7) (Fig. 3a, b). Grp94 levels varied considerably among the EL front limbs tested, but were typically higher than levels in the corresponding EL hind limbs although the difference was not statistically significant (*p* = 0.08; Fig. 3a, c and inset in Fig. 3c; *n* = 8 horses; Table 1). Dividing the EL front limb samples into PPID and obesity/regional adiposity risk factors yielded Grp94 levels that were not significantly different (*p* = 0.979; data summarized in Table 1).

### Immunofluorescence localizations of Grp78/BiP expressing cells within lamellar tissue

**Fig. 4:**
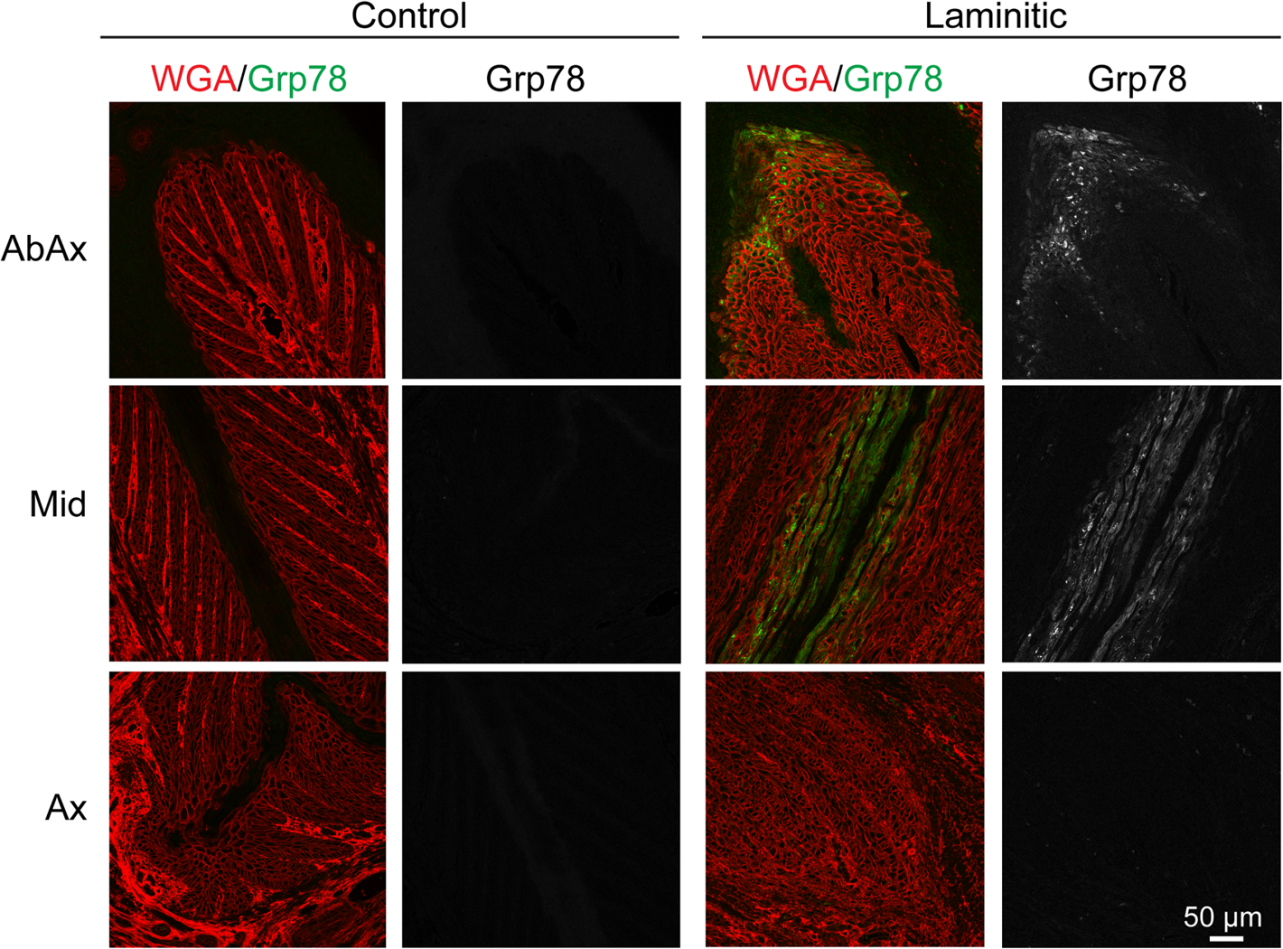
Localization of Grp78/BiP to suprabasal keratinocytes of the epidermal lamellae in laminitic tissue. Tissue sections were stained as described in Methods to localize Grp78/BiP (green) and wheat germ agglutinin (WGA; red) to outline cell plasma membranes (epidermis) and extracellular matrix (dermis). The Grp78/BiP channel is also shown separately in grayscale for each image. Representative images from Abaxial (AbAx), Middle (Mid) and Axial positions (Ax) are shown for control and laminitic samples. The control tissues showed no significant Grp78/BiP staining under the conditions used. In contrast, laminitic tissues showed bright Grp78/BiP expression in suprabasal epidermal keratinocytes located at the abaxial region and along the keratinized axis. Positive staining was absent from SELs in the axial region. All images are shown at the same magnification. Scale bar is 50 μm

**Fig. 5:**
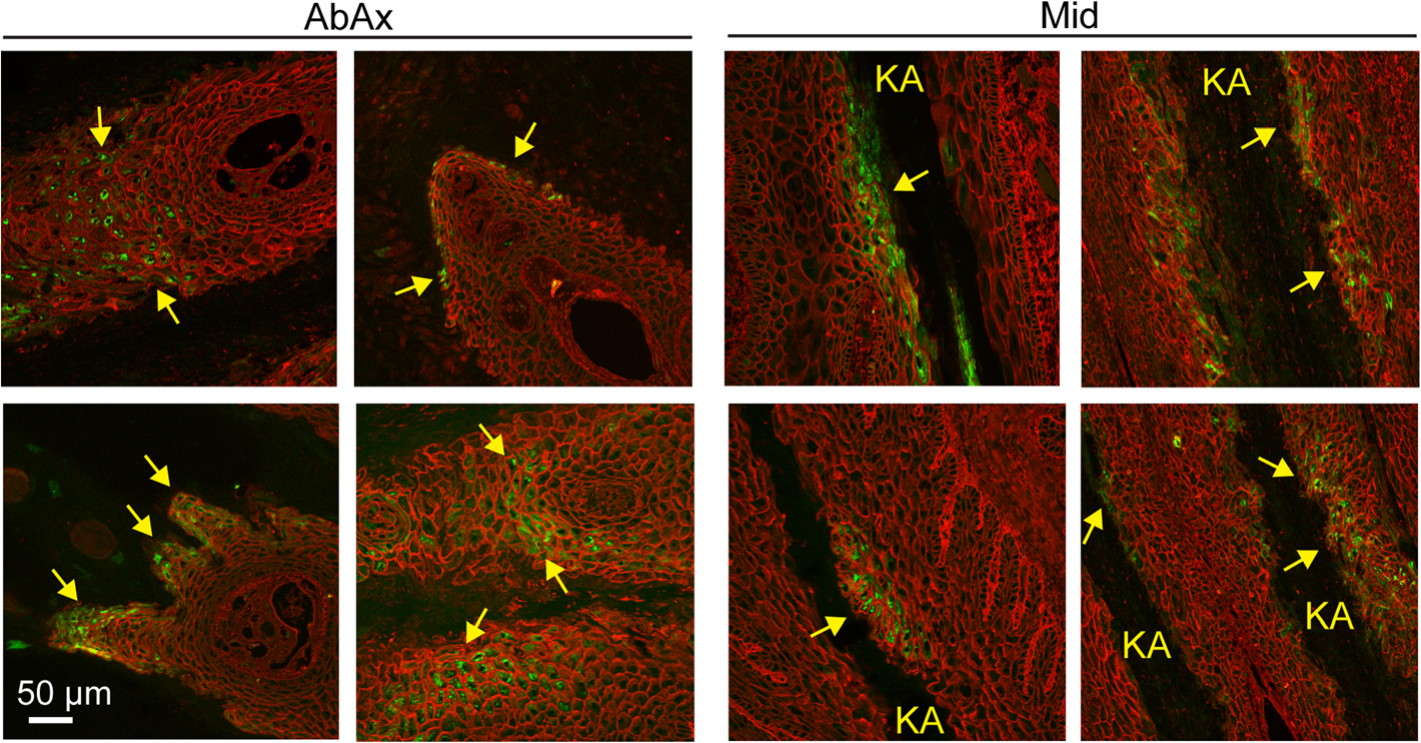
Positions of Grp78/BiP positive staining cells in lamellar tissue. All images are from laminitic samples. Grp78/BiP (green) and WGA (red) were localized as described in Methods. Images were selected to show the range of Grp78/BiP staining patterns observed in laminitic tissues. In the Abaxial region, Grp78/BiP expressing cells (yellow arrows) are found in multiple layers of suprabasal cells, or limited to a single layer of cells adjacent to the keratinized axis. In the middle region, Grp78/BiP positive cells (yellow arrows) are found adjacent to the keratinized axis (KA), often several cell layers deep. In some cases, positive cells are found along the length of the keratinized axis, while in other tissues expression is limited to groups of cells. All images are shown at the same magnification. Scale bar is 50 μm

While immunoblots provide an overall measure of protein expression level within the tissue, they do not address which cell type(s) show ER stress/UPR marker expression, or whether the variation in levels among the EL front limbs represents tissue-wide differences between samples or the number of individual cells possibly undergoing ER stress. Of the ER stress antibodies described above used for immunoblotting, one of the anti-Grp78/BiP antibodies proved useful for localization in formalin-fixed, paraffin-embedded tissue sections. Bright cytoplasmic staining, corresponding to high levels of Grp78/BiP expression, were detected in 10 of 12 sections of EL front tissue, while no sections from either control or EL hind tissues had detectable levels of Grp78/BiP (Fig. 4, Table 1). Although individual variation in number and location of positively stained cells existed among EL front limb samples, Grp78/BiP-positive cells were located as a single layer of suprabasal cells immediately adjacent to either the inner edge of the stratum medium or keratinized axis of the PEL, occasionally extending as multiple cell layers deep in these same locations (Figs. 4, 5). In the mid region of the PELs, cells expressing high levels of Grp78/BiP were often localized to small clusters of suprabasal cells immediately adjacent to the keratinized axis (Fig. 5), occasionally forming linear arrays of suprabasal cells extending along the length of the keratinized axis (Fig. 4). Many cells showed punctate Grp78/BiP localization, possibly representing protein aggregates within the ER. Other cells showed a more uniform protein distribution throughout the cytoplasm. Positively stained cells were absent from secondary epidermal lamellae (SELs) at the axial end of the PELs (Fig. 4).

## Discussion

The results described here demonstrate that several markers of ER stress/UPR are upregulated in natural cases of EL, compared to non-laminitic controls or corresponding hind limbs of horses with front limb laminitis. Specifically, spliced XBP1, a transcription factor, was detected by immunoblotting in most of the laminitic limbs examined. Two chaperones functioning in the ER lumen, Grp78/BiP and Grp94, were also expressed at higher levels in a significant number of laminitic samples compared to the control groups. Comparison of ER stress marker expression from EL front limbs to corresponding hind limbs showed greater expression in nearly all laminitic samples, although there was variation among samples and the difference in levels was modest for some cases. Potential factors contributing to variation could include differences in the stage of disease among individuals, the number of cells undergoing ER stress/UPR, or the time course of ER stress/UPR in cells at the time of euthanasia. Indeed, the two cases with moderate chronic-active laminitis (cases 73 and 165) showed less variation between front and hind limbs for each of the three markers examined compared to most of the severe chronic-active cases (Table 1). Additionally, differences in microanatomic sampling site in sections used for immunoblotting could potentially shift the ratio of dermal to epidermal tissue, generating artifactual variation among samples. However, given the abundance of epidermal cells relative to dermal cells per section at these sites, and that marker band intensities were normalized to intracellular proteins (tubulin or vinculin) primarily representative of epidermal tissues, the authors feel artifactual variation due to sampling site is less likely. Despite the variation among the EL front tissues, most samples showed the same general pattern of higher marker expression in the laminitic samples. Immunofluorescent localization of cells expressing high levels of Grp78/BiP supported immunoblot data with upregulation of Grp78/BiP only detected in the laminitic tissues (10/12 samples) and not in control or EL hind tissues (Table 1; Figs. 4, 5). Taken together, our results indicate that laminitic samples showed expression of ER stress/UPR markers. Cell stress may also contribute to sepsis-associated laminitis since experimental induction of laminitis by carbohydrate overload induced an upregulation of Grp94 mRNA [46].

Epidermal cells expressing high levels of Grp78/BiP were strikingly confined to laminitic front feet and to suprabasal cell layers immediately adjacent to the keratinized axis and hoof wall in abaxial to mid regions of the PEL and were absent from axial regions of the PEL (Figs. 4, 5). This suggests that ER stress/UPR is occurring in this specific cell type(s), but the significance of high expression of Grp78/BiP in these cells is not yet known. These cells may be at risk for apoptosis since prolonged activation of ER stress/UPR can lead to cell death by this pathway [26, 29, 30, 32]. Apoptosis in this region could compromise integrity of the lamellar epidermis and contribute to mechanical failure in laminitis. Consistent with this idea, we detected increased numbers of apoptotic/necrotic cells in the abaxial lamellar region of the laminitic limbs (see Additional file 5: Table S5A)), as has been noted by others for horses with hyperinsulinemia- and PPID-associated laminitis, especially in abaxial regions [7, 22]. Alternatively, cells expressing high levels of Grp78/BiP may reflect metaplastic and dyskeratotic cells; in preliminary experiments we find that Grp78/BiP positive keratinocytes are also metaplastic cells, as defined by increased Periodic acid-Schiff (PAS) staining of successive sections (our unpublished observations). Moreover, the distribution scores for hyperplasia, metaplasia, and orthokeratosis, all of which are features of the abnormal keratinization associated with lamellar wedge formation in chronic laminitis cases [4, 7, 47], were increased in the EL front feet. Dyskeratotic cells and abnormal keratinization were also noted in recent histopathology studies of naturally-occurring EL, particularly in abaxial regions [7, 22]. It is possible that ER stress/UPR contributes to the process of differentiation in these cells, similar to its previously noted participation in the normal differentiation of human skin suprabasal layer keratinocytes [48]. If ER stress/UPR positive cells are actively differentiating, this phenotype would be unique to laminitis and not to the normal tissue, and would therefore represent metaplastic differentiation. With the exception of the most proximal and distal regions of the hoof capsule, suprabasal cells in the epidermal lamellae undergo minimal cell proliferation [49] and differentiation [3] as part of normal tissue homeostasis, and we do not observe ER stress/UPR protein markers in lamellar tissue from the mid-dorsal region of the hoof capsules of non-laminitic or mildly laminitic limbs from control or laminitic horses. Consistent with a shift toward differentiation, the expression of p63, a marker of epithelial cell proliferative potential, is decreased in the abaxial basal SEL cells of horses with chronic laminitis and lamellar wedge formation [50]. Finally, ER stress and UPR within suprabasal cells at the abaxial region of PELs or adjacent to the keratinized axis could reflect a downstream consequence of loss of cell-cell or cell-extracellular matrix adhesions for cells lining the keratinized axis. Such a loss of adhesions should trigger increased synthesis of proteins needed for cell-cell or cell-matrix adhesions, possibly overloading the folding capacity of the ER and leading to ER stress and UPR. Whether ER stress/UPR is a primary or secondary event in EL pathogenesis, this pathway appears active in a subset of cells present at the time of fixation, and possibly reflects asynchronous activation of ER stress/UPR among the suprabasal cell populations in the chronic disease cases examined here. The involvement of ER stress/UPR in the acute stage of EL pathogenesis can be investigated using the experimental euglycemic-hyperinsulinemic clamp protocol [20, 21], which is currently the best available experimental EL model for assessing the toxic effect of high insulin levels on lamellar tissue, with the caveat that insulin levels may be much higher in the experimental model and disease onset may be more abrupt and severe than in natural EL [5].

It is of interest to note that both ER stress/UPR marker expression and laminitis pathology are increased in the front limbs, relative to the hind limbs, of the affected horses in this study. Possible explanations include either 1) ER stress occurs relatively late in laminitis pathogenesis and is downstream to mechanical failure of the lamellae, perhaps in association with abnormal keratinization and lamellar wedge formation or, 2) ER stress is an initiating event of laminitis and is more marked in the front limbs due to anatomical and physiological differences that cause epidermal lamellae of the front limbs to reside closer to a threshold for loss of cellular homeostasis, ER stress and laminitis. A difference between front and hind limbs that could alter this ER stress/laminitis threshold include the weight distribution disparity, with the front limbs bearing approximately 60% of body weight and the hind limbs bearing approximately 40% of body weight [51] which could impact the mechanics of digital suspension and lamellar perfusion, both of which impact cellular energy balance and ability to maintain homeostasis [52]. ER stress can result from either intrinsic factors, such as an overstimulation of protein synthesis by high insulin concentrations or a genetic mutation that causes protein misfolding, or extrinsic factors that impair ER function, such as decreased nutrient availability [30]. Although front and hind limbs should have equal exposure to the intrinsic factor, systemic hyperinsulinemia, in horses with insulin dysregulation associated with obesity or PPID, the front limbs may be more susceptible to ER stress due to increased energy demand and decreased nutrient delivery associated with limb loading [52]. The localization of Grp78/BiP to suprabasal cells in abaxial regions corresponds to the region with the most epidermal proliferation and abnormal keratinization in PPID- and hyperinsulinemia-associated laminitis [7, 22]. It is possible that ER stress is occurring downstream to other disease events and is marking metaplastic differentiation associated with lamellar wedge formation [50].

At least one of the ER Stress/UPR markers, Grp78/BiP, appears to localize predominantly to epidermal lamellar cells, but it is possible that other cell types have upregulated ER Stress/UPR signaling, either as a primary or secondary lesion of EL. In particular, ER stress was recently identified as a significant driver of diabetes-induced neuropathic pain in a mouse model [36]. Jones et al. [9] demonstrated that horses with chronic laminitis display peripheral nerve degenerative changes and neuropathic pain markers in the dorsal root ganglia cell bodies that supply the affected feet, consistent with neuropathic pain. Pharmacologic inhibition of soluble epoxide hydrolase, an arachidonate cascade enzyme that reduces levels of immunomodulatory epoxy fatty acids that modulate ER stress, can reverse pain behavior and ER stress in mice [36] and has shown promise for the treatment of intractable pain in equine laminitis cases [53]. Studies using samples from uniformly timed and earlier stages of hyperinsulinemia-induced experimental laminitis are needed to investigate the contribution of ER stress/UPR to the primary pathogenesis of EL and are currently underway. Further studies are needed to investigate the upstream control of ER stress and other cell stress pathways that mediate the toxic effects of high insulin levels on the lamellae.

The ER stress/UPR pathway is part of a larger circuit, with multiple possible targets to limit disease progression. ER stress/UPR can activate downstream pathways including inflammation [30, 36, 54, 55] and autophagy, where the latter can also act as an upstream trigger to cause ER stress/UPR [56]. ER stress/UPR and autophagy are both linked to activation of mammalian target of rapamycin complex 1 (mTORC1), an intermediate in a pathway recently demonstrated to be active in ponies treated with an experimental euglycemic-hyperinsulinemic clamp protocol to induce laminitis [57]. As discussed by Lane et al., rapamycin (an inhibitor of mTORC1) and other inhibitors acting on the mTORC1 pathway are potential therapeutics for treatment and prevention of laminitis. Inhibitors of the mTORC1 pathway decrease ER stress by increasing autophagy and allowing cells to dispose of misfolded proteins [58]. Although promising, rapamycin has shown toxicity in human trials [59, 60] and some authors have suggested that rapamycin will be more effective in combination therapies [59]. Small molecules have been identified which either modulate protein function within the ER stress pathway or enhance the protein folding capacity within the ER lumen to relieve ER stress and are potentially useful in the treatment of multiple human diseases [32]. These pharmacological approaches to ER stress amelioration include drugs that have been used in equine medicine for the treatment of equine metabolic syndrome (metformin) and musculoskeletal inflammation (dimethyl sulfoxide, DMSO) [61, 62], as well as the soluble epoxide hydrolase inhibitors used to treat neuropathic pain [53]. Additional drugs that are being investigated for use in the treatment of human metabolic syndrome reduce ER stress by increasing endogenous chaperone expression (GLP-1 receptor agonists) or by serving as chemical chaperones (4-phenylbutyric acid and tauroursodeoxycholic acid (TUDCA)) [63]. Metformin, GLP-1 receptor agonists, and chemical chaperones could have a dual effect on both the underlying insulin dysregulation and hyperinsulinemia that triggers EL, and on laminitis pathogenesis at the lamellar tissue level [41, 59]. DMSO, long used for its anti-inflammatory properties, acts as a chemical chaperone through to its detergent effect and ability to coat proteins in the ER and mask hydrophobic patches on unfolded proteins, which increases their secretion and relieves ER stress [63]. It is possible that such drugs also may be useful in treating laminitis in the horse, either alone or in combination with inhibitors of autophagy and/or the mTORC1 pathway since these pathways all likely fall into a larger circuit.

## Conclusions

In summary, our data support the hypothesis that the ER stress/UPR pathway is active in naturally-occurring equine EL, which possibly links this disease with a number of human diseases where ER stress/UPR activation is thought to make a significant contribution to disease progression [25–36]. Identification of shared cellular- and molecular-based disease mechanisms common to equine laminitis and human diseases will leverage the extensive progress in understanding these human diseases and facilitate the identification of possible therapeutic interventions amenable to treatment of equine laminitis.

## Methods

### Tissue samples

A cohort of 12 EL cases of bilateral forelimb laminitis and 8 age-matched controls (Table 1) were obtained from the Laminitis Discovery Database, a tissue repository housed in the Galantino-Homer laboratory at the University of Pennsylvania School of Veterinary Medicine, New Bolton Center [40]. Samples available in the repository were collected from horses at the time of euthanasia by pentobarital and phenytoin overdose according to procedures approved by the University of Pennsylvania IACUC and include 14 horses donated for teaching and research and 6 horses submitted for necropsy. Tissues containing lamellae, 1–2 mm of adjacent hoof wall, and sub-lamellar dermis were dissected immediately after euthanasia from the mid-dorsal region of the hoof, as described previously [4].

EL cases (12 horses) and non-laminitic controls (8 horses) were a mix of males and females of several breeds, aged 6–33 years, as detailed in Table 1. EL cases were defined by: 1) the clinical expression of signs compatible with chronic-active front foot laminitis at the time of euthanasia, as reported by the attending veterinarian or owner, and confirmed by gross pathology and histopathology as described below (see Additional files 1, 2, 3, 4, 5, 6, 7 and 8), and 2) the presence of one or more of the following phenotypic indicators of EL: antemortem clinical diagnosis by a veterinarian of PPID or obesity (body condition score > or = 7) and/or regional adiposity (cresty neck score ≥ 3) [16, 41, 42, 64, 65], pituitary adenoma lesions as confirmed by an anatomic veterinary pathologist (J.B.E.), history of hyperinsulinemia, elevated adrenocorticotropic hormone (ACTH), or abnormal glucose tolerance test or ACTH stimulation test, and (3) lack of other laminitis risk factors at initial diagnosis, such as sepsis, carbohydrate overload, or non-weight-bearing lameness. The cohort of cases examined included 6 horses with PPID and 6 with obesity and/or regional adiposity. Eight age-matched controls were euthanized primarily for lameness due to non-laminitic orthopedic disease (7 horses) or infertility (1 horse) and showed few gross or histological lesions compatible with laminitis (Table 1 and Tables A1-A8 (see Additional files 1, 2, 3, 4, 5, 6, 7 and 8). Although none of the EL cases had hind foot laminitis reported in their clinical history, or had lesions compatible with laminitis on gross postmortem exam, four of the ten hind feet used for these studies, as well as an EL case hind foot that was not used in the study (case #140), did have histological lesions consistent with laminitis and the remaining six hind feet, plus an unused hind foot from EL case #90, did not. The four unaffected and six less affected hind feet were included in the study as a separate group from control and affected feet to compare the relative effects of moderate to severe laminitis (front feet) to less affected hind feet from the same horses (Table 1; Tables A1-A8 (see Additional files 1, 2, 3, 4, 5, 6, 7 and 8).

### Antemortem clinical characterization and postmortem gross/histological evaluation

Each foot was scored for antemortem presence and duration (in days) of clinical signs compatible with laminitis, as well as gross and histologic postmortem lesions compatible with four laminitis categories (non-laminitic, sub-clinical, moderate chronic-active, and severe chronic-active), as described previously [4] with modifications detailed in Additional Methods and Tables A1-A8 (see Additional files 1, 2, 3, 4, 5, 6, 7, 8 and 11) and summarized in Table 2. The four categories were: Non-laminitic (no clinical signs of laminitis, a normal or minimal gross pathology score (gross pathology score 1) and no histological changes compatible with laminitis); Sub-clinical (no clinical signs of laminitis, gross pathology score 1, but histological lesions compatible with lamellar damage are present); Moderate laminitis (more than 7 days duration of active clinical signs of laminitis, gross pathology score of 2 or 3, and histological lesions compatible with laminitis); Severe laminitis (more than 7 days duration of clinical signs of laminitis, gross pathology score of 4, and histological lesions compatible with laminitis). Gross pathology was assessed from direct observations and photographic images captured immediately after mid-sagittal sectioning of the foot, as described in detail in Additional Methods reported in Table A1 (see Additional files 1 and 11). Quantitative and qualitative histology was assessed as previously described on formalin-fixed/paraffin-embedded lamellar tissue samples that were sectioned and stained with hematoxylin and eosin stain and with Periodic acid-Schiff-hematoxylin stain, with modifications as follows [4]. Quantitative lesions compatible with laminitis were assessed from light microscopic images of each section using ImageJ software (NIH), as detailed in Additional Methods and reported in Tables A2 and A3 (see Additional files 2, 3 and 11). Measurements were averaged from five randomly selected PELs or SELs at three locations relative to the axis of the digit (abaxial, middle and axial) within the image. Qualitative lesions compatible with laminitis were scored for presence or absence of each lesion with its distribution (e.g. focal, multifocal, regional and global), as detailed in Additional Methods and reported in Tables A4-A8 (see Additional files 4, 5, 6, 7, 8 and 10). Measurements included: PEL length, SEL length, mid-SEL width, angle between axis of SEL and axis of PEL, keratinized axis displacement (i.e., distance from the axial end of the keratinized axis to axial end of its respective PEL), and SDL displacement (i.e., distance from the tip of the secondary dermal lamella (SDL) to the lateral edge of the keratinized axis). With the exception of SDL displacement, these measurements were localized as previously described [44]. The qualitative histology score was generated by adding distribution scores for the following lesions as previously described [4], with some modifications, and as detailed in Additional Methods (see Additional file 11): Pleomorphic lamellar epidermal basal cell nuclear morphology, basement membrane lesions, displacement or detachment of secondary epidermal lamellae from the keratinized axes of the primary epidermal lamellae, abnormal epidermal lamellar morphology (detached epidermal islands, elongated, broken, necrotic, or merged secondary epidermal lamellae), epidermal cell lesions (hyperplasia, metaplasia, orthokeratosis, or vacuolated cells), dermal spindle cell hypertrophy, expanded dermal interstitial space or loss of dermal connective tissue, inflammatory cell infiltration, vascular lesions, and increased apoptotic/necrotic and mitotic figure cell counts. Because an overall qualitative histopathology score of 30 or above was associated with increased scores for histological lesions previously associated with EL [4, 7, 22], including lamellar epidermal basal cell pleomorphism, thickened or fragmented basement membrane, SDL retraction, SEL morphology changes, epidermal hyperplasia, metaplasia and/or orthokeratosis, increased apoptotic/necrotic cells, and/or increased mitotic figures, feet with a score of 30 or above were defined to have histological lesions consistent with either laminitis or subclinical lamellar damage, and feet scoring 29 or below were defined as non-laminitic. Data were analyzed by Kruskal-Wallis ANOVA on ranks followed by all pairwise multiple comparison using Dunn’s Method, unless otherwise noted (SigmaStat version 3.5; Systat Software, Inc.)

### Human cell line

As a positive control for immunoblots and to test antibody specificity, lysates were prepared from the human Hela cell line as described previously [66]. Where noted, Hela cells were incubated for 24 h in either Brefeldin A (BFA; 3.6 μM; Sigma-Aldrich) or tunicamycin (TM; 2 μg/ml; Sigma-Aldrich) to induce ER stress prior to lysate preparation [45].

### Immunoblotting

Frozen tissue was prepared for immunoblotting by pulverizing in liquid nitrogen and extracting proteins in SDS/glycerol buffer (supplemented with 1 mM PMSF and Halt Protease Inhibitor Cocktail (ThermoScientific)). The extract was heated to 100 °C for 5 min and cleared by pelleting unsoluble material at 13,000 x g at 4 °C for 20 min. The supernatants were collected and protein concentration measured using a Pierce micro BCA assay kit with a BSA standard (Bio Rad). Samples were stored at − 80 °C. After adding loading dye and ß-mercaptoethanol, samples were heated to ~ 100 °C for 5 min and 20 μg of total protein loaded per well of 10% acrylamide gels. Proteins were transferred to PVDF membranes (0.2 μm Bio Rad) using a semi-dry transfer (Bio Rad Trans-Blot Turbo). Membranes were incubated in blocking buffer (5% nonfat milk in Tris buffered saline (TBS) supplemented with 0.1% Tween 20) for 1–2 h at room temperature. Membranes were incubated in primary antibodies overnight at 4 °C (XBP1 or Grp78/BiP antibodies diluted in 20% blocking buffer/80%TBS-tween; Grp94 diluted in blocking buffer). After three 10 min washes in TBS-tween at room temperature, membranes were then incubated in anti-rabbit (diluted 1:20,000, BD Biosciences) or anti-mouse (diluted 1:10,000, Abcam) IgG conjugated to HRP and incubated for 1 h at room temperature. After three 10 min washes in TBS-tween, immunoreactive bands were developed with enhanced chemilumincence (ECL prime for most antibodies, standard ECL for tubulin; GE Healthcare). Primary antibodies included: rabbit polyclonal anti-XBP1 (1:2000; Thermo Scientific, PA5–25010), rabbit monoclonal anti-Grp78/BiP (1:1000; Cell Signaling Technology clone C50B12), rabbit polyclonal anti-Grp78/BiP (1:1000; AbCam, ab21685), and rabbit monoclonal anti-Grp94 (1:1000; Cell Signaling Technology clone D6X2Q). Membranes were reprobed with rabbit anti-vinculin (1:1000; Cell Signaling clone E1E9V) or mouse monoclonal anti-alpha tubulin (1:5000; Sigma, clone B-5-1-2) as loading controls.

To compare samples between blots, films were scanned, the black/white scale was inverted, and band intensities were measured in Image J. Band intensities were normalized based on loading controls (tubulin or vinculin). To allow comparison between blots, a single sample, 116 LF (XBP1s or Grp94) or 134 RF (Grp78/BiP), was included in all blots used for semi-quantitations and the normalized intensity of this band was set to a value of 100 arbitrary units. The intensities of all other bands on each film were scaled accordingly. This normalization procedure allowed us to compare data from multiple experiments. Box plots were graphed in Kaleidagraph (version 4.5; Synergy Software). In these plots the central box encompasses 50% of the data, the median value is shown by the horizontal line within the box, lines extending from the top or bottom of the box extend to the minimum and maximum values, with the exception of outliers which are displayed as individual points. Means and standard deviations were calculated using Excel (version 15.36). Values from control and laminitic front limbs were compared using unpaired t-test with unequal variance, calculated using Kaliedagraph (version 4.5). Values measured in laminitic front limbs were compared to those from the hind limbs of the same horses by paired t-test using Kaliedagraph (version 4.5).

### PCR amplification

Primers were designed to flank the splice site in the XBP1 mRNA (*Equus caballus* reference sequence XM_014742035.1). Forward primer 5’ TTACGCGAGAAAACTCATGGCC 3′ and reverse primer 5’ GGGTCCAAGTTGAACAGAATGC 3′ amplify a 281 base pair fragment (unspliced) or a 255 base pair fragment (spliced). Total RNA was isolated from lamellar samples and the quality of the RNA confirmed by A260/A280 ratios of 2.0 for all samples tested. The RNA was reverse transcribed to cDNA (TaqMan reverse transcription reagents kit; Applied Biosystems) according to the manufacturer’s instructions. cDNA was used as template for PCR amplification (Taq DNA polymerase, New England Biolabs). Amplimers were separated on 3% agarose gels containing ethidium bromide. The quality of the RNA was confirmed by amplifying a region of *E. caballus* RACK1 (NM_001242446.1) spanning an intron/exon boundary. Forward primer 5’ CAGGGATGAGACCAACTACG 3′ and reverse primer 5’ ATGCCACACTCAGCACATC 3′ amplify a 200 bp sequence from RNA and a 741 bp sequence from genomic DNA. The 200 bp product was the only amplimer detected in all samples (not shown). Gels were imaged using a ChemiDoc MP imaging system (BioRad) and Image Lab software (version 5.1; BioRad). Images were exported as tiff files and the black/white scale inverted in Photoshop (version CS3; Adobe) for clarity.

### Immunfluorescence

Paraffin-embedded 6 μm thick sections of formalin-fixed tissues were used for immunofluorescent localization of Grp78/BiP (Cell Signaling Technology clone C50B12). Most tissue sections examined came from the same limb as that used for immunoblotting, although a few came from a second laminitic front limb from the same horse (see Table 1). The choice of limb/tissue block used for immunofluorescence was made based on the quality of the tissue sections and/or availability of sections in hand, and therefore tissues examined were selected without bias. Tissue sections were oriented transverse to the axis of the limb and include the entire abaxial, middle, and axial regions of the primary epidermal lamellae as well as the sub-lamellar dermal tissue located between the distal phalanx and epidermal lamellae. De-paraffinization, rehydration and antigen retrieval were conducted as described previously [67]. After incubation in Background Buster (Innovex Biosciences), slides were incubated in rabbit monoclonal anti-Grp78/BiP (1:100; Cell Signaling Technology clone C50B12) for 50 min, washed 3 × 10 min in PBS, incubated in goat anti rabbit IgG-Alexa 488 (1:100) for 40 min, washed 1 × 10 min, incubated in rhodamine tagged wheat germ agglutinin (12.5 μg/ml; Vector Laboratories; [68]) for 15 min, washed 2 × 10 min in PBS and finally mounted in Vectashield (Vector Laboratories). Slides were incubated with antibodies or WGA at room temperature in a humid chamber; PBS washes were on ice. The rhodamine-tagged WGA, a lectin, was used as a counterstain to label extracellular matrix and plasma membranes and facilitate the identification of lamellar microanatomy [68].

Sections were imaged using a Zeiss 880 confocal microscope system including a Zeiss Axio Observer inverted microscope stand and 25X PlanApo objective (0.8 NA), controlled by Zen software (version 2.1). Laser power (run at 1% for 488 nm laser line and 2.8% for 561 nm line) and gain were nearly identical for all experiments. Images were collected using a 0.7x zoom to maximize image field size at a pixel depth of 1024 × 1024 and with 4 frame averaging to reduce noise. Images were exported as tiff files, adjusted in Photoshop (version CS3; Adobe) by linearly adjusting brightness of each image and applying identical settings to all images. The images were assembled into figures in Illustrator (version CS5; Adobe). Grp78/BiP localization was evaluated in abaxial, mid, and axial anatomical regions of the primary epidermal lamella (PEL) that were defined relative to the central axis of the digit.

## Additional files

Additional file 1: **Table S1.** Sample data summary of duration, gross pathology severity score, and Stratum Internum-Corium Measurements. The laminitis duration, gross pathology severity score based on the presence of lesions compatible with laminitis, and stratum internum-corium measurements (SICM) for the front and hind feet of horses with Endocrinopathy-Associated Laminitis (EL) and front feet from control horses used in the present study. (DOCX 19 kb)

Additional file 2: **Table S2.** Quantitative Histology Measurements I: PEL length, KA width and displacement from axial SEL tip and SDLs. This table summarizes measurements of primary epidermal lamella (PEL) length, PEL Keratinized axis (KA) width, Displacement of axial secondary epidermal lamellae (SEL) from the KA (KA Disp), and distance between KA and secondary dermal lamella (KA-SDL) of mid-dorsal lamellae from the front and hind feet of horses with Endocrinopathy-Associated Laminitis (EL) and front feet from control horses (Means of 5 measurements + Standard Deviations). (DOCX 22 kb)

Additional file 3: **Table S3.** Quantitative Histology Measurements II: SEL length, width and angle. This table summarizes measurements of the mean length, width and angle of secondary epidermal lamellae (SEL) of mid-dorsal lamellae from the front and hind feet of horses with Endocrinopathy-Associated Laminitis (EL) and front feet from control horses. (DOCX 23 kb)

Additional file 4: **Table S4.** Overall qualitative histopathology severity score and histopathology lesion distribution scores. This table summarizes qualitative histopathology scores from the evaluation of H&E and PASH stained lamellar tissue sections by light microscopy, as described in the Supplemental Methods section. The overall (additive) histopathology score is listed for each limb and is derived from distribution scores for specific types of lesions that are also listed on this table (Lamellar Epidermal Basal Cell Pleomorphism, Basement Membrane, KA-axial SEL displacement, SEL Morphology, Epidermal Pathology, Dermal Spindle Cell Hypertrophy, Dermal Interstitial Pathology, White Blood Cells, and Vascular Pathology) and Table A5 (Lamellar Apoptotic/Necrotic and Mitotic Figure distribution scores). (DOCX 23 kb)

Additional file 5: **Table S5.** Lamellar apoptotic/necrotic and mitotic figure distribution scores. Table summarizing apoptotic/necrotic cell and SEL mitotic figure distribution scores for samples used in the current study. (DOCX 20 kb)

Additional file 6: **Table S6.** Qualitative Lamellar Basement Membrane histopathology lesion distribution scores. Table summarizing individual basement membrane (BM) histopathological lesion distribution scores that contributed to the BM pathology score reported in Table A4 for samples used in the current study, including distributions of thickened BM, fragmented BM, BM detached at SEL tips, SDL retraction, and completely detached BM. (DOCX 19 kb)

Additional file 7: **Table S7.** Qualitative lamellar epidermal histopathology lesion distribution scores. Table summarizing individual SEL morphology and epidermal differentiation lesions that contributed to the SEL Morph and Epid Path distribution scores reported in Table A4. Individual SEL morphology lesions include axial PEL blunting, SEL islands, abnormal SEL shape, merged SELs, and necrotic SELs. Individual epidermal differentiation lesions include hyperplasia, metaplasia, and orthokeratosis. (DOCX 21 kb)

Additional file 8: **Table S8.** Qualitative lamellar leukocyte and vascular histopathology lesion distribution scores. Table summarizing individual leukocyte type and vascular histopathology lesion distribution scores that contributed to the WBC and Vascular Path distribution scores reported in Table A4. Individual leukocyte types include lymphocytic, histiocytic, plasmacytic, polymorphonuclear leukocytic, and hemosiderophagocytic. Individual vascular pathology lesions include thromboemboli, perivascular inflammation, endothelial hypertrophy, and vasodilation. (DOCX 21 kb)

Additional file 9: Additional validation of immunoblot band intensity measurements and comparison of two commercial antibodies to Grp78/BiP. The figure shows two plots to provide additional validation of the immunoblot band intensity measurements. (DOCX 21 kb)

Additional file 10: Additional validation of immunoblot band intensity measurements and comparison of two commercial antibodies to Grp78/BiP. Figure legend for additional file 9. (DOCX 23 kb)

Additional file 11: Methods are described for quantitative histology measurements and qualitatitive histopathology distribution and severity scoring for the cases used in the current study. (DOCX 26 kb)

## Abbreviations

BiP: Binding immunoglobulin protein (also known as Grp78)
EL: Endocrinopathic laminitis
EMS: Equine metabolic syndrome
ER: Endoplasmic reticulum
Grp78: 78 kD Glucose-regulated Protein (also known as BiP)
Grp94: 94 kD Glucose-regulated protein
IRE-1: Inositol requiring enzyme 1
OB/RA: Obesity and regional adiposity
PEL: Primary epidermal lamellae
PPID: Pituitary pars intermedia dysfunction
SEL: Secondary epidermal lamellae
UPR: Unfolded protein response
XBP1s: Spliced X-box binding protein 1
