# Supplementary material for: Detection of endoplasmic reticulum stress and the unfolded protein response in naturally-occurring endocrinopathic equine laminitis
Source: BMC Vet Res. 2019 Jan 10;15:24. doi: 10.1186/s12917-018-1748-x (PMC6327420; doi:10.1186/s12917-018-1748-x)
Supplement: Supplementary file 1 — Table S1. Sample data summary of duration, gross pathology severity score, and Stratum Internum-Corium Measurements. The laminitis duration, gross pathology severity score based on the presence of lesions compatible with laminitis, and stratum internum-corium measurements (SICM) for the front and hind feet of horses with Endocrinopathy-Associated Laminitis (EL) and front feet from control horses used in the present study. (DOCX 19 kb) [file 12917_2018_1748_MOESM1_ESM.docx]

| **Table A1. Sample data summary of duration, gross pathology severity score, and Stratum Internum-Corium Measurements.** | | | | | |
| --- | --- | --- | --- | --- | --- |
| **ID** | **Duration (days)** | **Gross Pathology Severity Score** | **SICM (mm)** | | |
| **Control** |  |  | **Proximal** | **Middle** | **Distal** |
| 61 RF | 0 | 1 | 6 | 6 | 6 |
| 92 LF | 0 | 1 | 6 | 6 | 6 |
| 102 LF | 0 | 1 | 5 | 5 | 5 |
| 110 LF | 0 | 1 | 6 | 6 | 7 |
| 111 LF | 0 | 1 | 7 | 6 | 6 |
| 113 LF | 0 | 1 | 6 | 6 | 6 |
| 114 LF | 0 | 1 | 6 | 5.5 | 6 |
| 129 RF | 0 | 1 | 5 | 5 | 6 |
| **Mean + SD:** |  | **1** | **5.9 + 0.6** | **5.7 + 0.5** | **6.0 + 0.5** |
| **EL Front** |  |  |  |  |  |
| 63 RF | 30 | 4 | 6 | 8 | 8 |
| 63 LF | 30 | 4 | 6.5 | 8 | 9 |
| 73 LF | 365 | 3 | 7 | 6 | 7 |
| 75 RF | 3650 | 4 | 11 | 20 | 30 |
| 75 LF | 3650 | 4 | 9 | 15 | 25 |
| 90 LF | 1825 | 4 | 5 | 7 | 8 |
| 101 RF | 90 | 4 | 6 | 13 | 20 |
| 104 RF | >365 | 4 | 7 | 10 | 25 |
| 109 LF | >365 | 3 | 5 | 6.5 | 7 |
| 116 LF | 60 | 4 | 7 | 10 | 12 |
| 116 RF | 60 | 3 | 4.5 | 8 | 10 |
| 134 RF | 1095 | 4 | 10 | 13 | 17 |
| 134 LF | 1095 | 4 | 10 | 14 | 18 |
| 140 LF | 365 | 4 | 6 | 8 | 10 |
| 141 LF | >60 | 4 | 8 | 13 | 18 |
| 141 RF | >60 | 3 | 5 | 8 | 10 |
| 165 LF | >120 | 4 | 6.5 | 7 | 12 |
| **Mean + SD:** |  | **3.6 + 0.8**** | **7.0 + 1.9*** | **10.3 + 3.8**** | **14.5 + 7.2**** |
| **EL Hind** |  |  |  |  |  |
| 63 LH | 0 | 1 | 5 | 5 | 5 |
| 73 LH | 0 | 1 | 6 | 5 | 5 |
| 75 RH | 0 | 1 | 5 | 5 | 5 |
| 101 LH | 0 | 1 | 5 | 4.5 | 5 |
| 104 RH | 0 | 1 | 5 | 5.5 | 5.5 |
| 109 RH | 0 | 1 | 4 | 4 | 5 |
| 116 RH | 0 | 1 | 4 | 4 | 5 |
| 134 RH | 0 | 1 | 5 | 5 | 6 |
| 141 RH | 0 | 1 | 4 | 4.5 | 5 |
| 165 LH | 0 | 1 | 6 | 6 | 6 |
| **Mean + SD:** |  | **1** | **4.9 + 0.7** | **4.9 + 0.6** | **5.3 + 0.4** |

**Duration** of clinical signs reported by owner or referring veterinarian in days or approximate days (as indicated by greater than symbol (>)), **Gross Pathology Severity Score** (1-4) as described in the Supplemental Methods (Additional file 11), and **SICM** measurement in mm from the dorsal surface of the distal phalanx to inner edge of the hoof wall/stratum medium, encompassing the lamellae/stratum internum and corium measurement, at proximal, middle, and distal locations along the hoof, as described in the Methods section.

**ID**: Identification of individual feet evaluated; **Control**: Non-laminitic or mildly/subclinically affected (control) front feet; **EL Front**: Moderately to severely affected front feet from horses with endocrinopathic laminitis; **EL Hind**: Non-laminitic or mildly/subclinically affected hind feet from horses with endocrinopathic laminitis; **LF:** Left Front foot; **LH:** Left Hind foot; **RF:** Right Front foot; **RH:** Right Hind foot.

The means and standard deviations (SD) are shown below individual foot values for each group for Gross Score and SICM measurements. Since data were not normally distributed, scores and measurements compared between groups using Kruskal-Wallis One Way Analysis of Variance (ANOVA) on Ranks followed by all pairwise multiple comparison using Dunn’s Method.

*Differs from EL Hind, but not Control (P<0.05).

**Differs from EL Hind and Control (P<0.05).
