# Supplementary material for: Detection of endoplasmic reticulum stress and the unfolded protein response in naturally-occurring endocrinopathic equine laminitis
Source: BMC Vet Res. 2019 Jan 10;15:24. doi: 10.1186/s12917-018-1748-x (PMC6327420; doi:10.1186/s12917-018-1748-x)
Supplement: Supplementary file 2 — Table S2. Quantitative Histology Measurements I: PEL length, KA width and displacement from axial SEL tip and SDLs. This table summarizes measurements of primary epidermal lamella (PEL) length, PEL Keratinized axis (KA) width, Displacement of axial secondary epidermal lamellae (SEL) from the KA (KA Disp), and distance between KA and secondary dermal lamella (KA-SDL) of mid-dorsal lamellae from the front and hind feet of horses with Endocrinopathy-Associated Laminitis (EL) and front feet from control horses (Means of 5 measurements + Standard Deviations). (DOCX 22 kb) [file 12917_2018_1748_MOESM2_ESM.docx]

| **Table A2: Quantitative Histology Measurements I: PEL length, KA width and displacement from axial SEL tip and SDLs.** | | | | | | |
| --- | --- | --- | --- | --- | --- | --- |
| **ID** | **PEL Length (mm)** | **KA Width (μm)** | **KA Disp (mm)** | **KA-SDL Distance (μm)** | | |
| **Control** |  |  |  | **Abaxial** | **Middle** | **Axial** |
| 61 RF | 3.6 + 0.1 | 36.1 + 8.8 | 0.27 + 0.07 | 13.9 + 4.5 | 14.5 + 4.6 | 16.1 + 5.0 |
| 92 LF | 3.9 + 0.1 | 38.6 + 4.7 | 0.10 + 0.02 | 10.3 + 2.7 | 14.0 + 6.7 | 29.0 + 21.5 |
| 102 LF | 3.4 + 0.3 | 27.5 + 11.3 | 0.26 + 0.10 | 16.8 + 5.7 | 7.5 + 6.0 | 15.9 + 6.9 |
| 110 LF | 3.2 + 0.1 | 40.5 + 7.1 | 0.28 + 0.05 | 11.5 + 3.2 | 16.9 + 3.0 | 20.2 + 3.7 |
| 111 LF | 3.5 + 0.2 | 36.2 + 8.9 | 0.22 + 0.08 | 10.3 + 3.0 | 8.8 + 4.6 | 21.0 + 10.8 |
| 113 LF | 3.3 + 0.4 | 57.8 + 5.7 | 0.11 + 0.08 | 10.6 + 4.6 | 11.2 + 1.6 | 9.8 + 4.8 |
| 114 LF | 3.0 + 0.3 | 27.0 + 4.3 | 0.44 + 0.11 | 7.2 + 3.3 | 10.0 + 3.4 | 12.6 + 4.6 |
| 129 RF | 3.6 + 0.4 | 45.2 + 5.9 | 0.24 + 0.05 | 34.4 + 13.4 | 14.4 + 3.3 | 29.6 + 11.3 |
| **Mean + SD:** | **3.4 + 0.4** | **38.6 + 11.6** | **0.24 + 0.12** | **14.4 + 9.8** | **12.2 + 5.1** | **19.3 + 11.5** |
| **EL Front** |  |  |  |  |  |  |
| 63 RF | 5.0 + 0.3 | 39.3 + 13.0 | 1.51 + 0.10 | 54.0 + 19.2 | 46.1 + 20.1 | 37.4 + 10.9 |
| 63 LF | 4.9 + 0.1 | 41.9 + 16.1 | 1.09 + 0.29 | 47.1 + 12.6 | 57.0 + 15.2 | 118.6 + 43.7 |
| 73 LF | 4.2 + 0.3 | 35.8 + 9.1 | 0.31 + 0.05 | 17.7 + 3.9 | 25.4 + 2.2 | 23.3 + 7.9 |
| 75 RF | NA | NA | NA | NA | NA | NA |
| 75 LF | 8.2 + 0.6 | 31.0 + 8.4 | 5.20 + 0.74 | 136.8 + 40.5 | 52.5 + 31.0 | 112.8 + 34.7 |
| 90 LF | 5.8 + 0.6 | 19.3 + 5.8 | 3.00 + 0.54 | 30.7 + 11.4 | 31.6 + 17.9 | NA |
| 101 RF | 8.2 + 0.2 | 62.4 + 37.4 | 2.76 + 1.07 | 110.2 + 12.5 | 170.6 + 90.8 | 77.4 + 27.8 |
| 104 RF | 7.4 + 0.5 | 89.7 + 31.4 | 1.52 + 0.72 | 47.2 + 6.2 | 37.6 + 19.1 | 20.4 + 4.9 |
| 109 LF | 5.2 + 0.2 | 48.8 + 20.7 | 1.76 + 0.27 | 20.5 + 10.7 | 17.2 + 3.7 | 31.9 + 12.8 |
| 116 LF | 8.2 + 0.3 | 86.7 + 11.7 | 1.25 + 0.31 | NA | NA | 831.9 + 223.3 |
| 116 RF | 2.7 + 0.6 | 48.9 + 27.7 | 0.91 + 0.31 | 57.0 + 26.4 | 111.1 + 39.7 | 191.9 + 128.4 |
| 134 RF | 6.4 + 0.3 | 14.7 + 1.6 | 1.28 + 0.31 | 25.0 + 23.5 | 200.6 + 53.8 | 88.9 + 47.3 |
| 134 LF | 8.8 + 0.4 | 124.7 + 31.4 | 2.14 + 0.85 | 51.8 + 18.2 | 63.5 + 24.5 | 57.1 + 20.5 |
| 140 LF | 5.0 + 0.2 | 102.0 + 43.7 | 0.61 + 0.11 | 57.8 + 16.8 | 48.1 + 20.6 | 36.6 + 10.3 |
| 141 LF | 4.4 + 1.6 | 68.5 + 41.8 | 1.63 + 0.83 | 55.8 + 6.5 | 77.0 + 24.6 | 81.3 + 23.5 |
| 141 RF | 6.1 + 0.4 | 111.7 + 33.8 | 1.81 + 0.23 | 55.2 + 14.9 | 55.1 + 32.1 | 132.1 + 79.4 |
| 165 LF | 4.7 + 0.5 | 31.8 + 15.2 | 1.15 + 0.38 | 35.9 + 8.5 | 30.5 + 13.6 | 35.3 + 14.0 |
| **Mean + SD:** | **5.9 + 1.8**** | **59.8 + 40.2** | **1.74 + 1.23**** | **54.0 + 35.0**** | **68.3 + 60.3**** | **115.6 + 188.9**** |
| **EL Hind** |  |  |  |  |  |  |
| 63 LH | 3.0 + 0.1 | 26.6 + 8.1 | 0.32 + 0.10 | 20.4 + 7.0 | 33.7 + 7.0 | 25.3 + 11.1 |
| 73 LH | 3.4 + 0.0 | 51.8 + 7.3 | 0.18 + 0.06 | 28.9 + 6.1 | 13.8 + 3.8 | 14.7 + 5.1 |
| 75 RH | 2.6 + 0.1 | 36.5 + 10.2 | 0.63 + 0.08 | 10.3 + 4.4 | 11.2 + 5.3 | 15.8 + 7.4 |
| 101 LH | 2.8 + 0.2 | 40.9 + 6.8 | 0.46 + 0.07 | 22.7 + 6.5 | 22.7 + 4.2 | 27.3 + 7.6 |
| 104 RH | 2.8 + 0.1 | 30.2 + 6.4 | 0.28 + 0.15 | 24.7 + 3.2 | 26.4 + 5.9 | 33.5 + 5.6 |
| 109 RH | 3.3 + 0.1 | 40.5 + 8.6 | 0.62 + 0.22 | 16.8 + 4.2 | 16.9 + 5.5 | 30.0 + 8.4 |
| 116 RH | 3.2 + 0.2 | 25.8 + 9.0 | 0.42 + 0.15 | 12.8 + 1.5 | 7.4 + 5.3 | 16.8 + 4.3 |
| 134 RH | 3.2 + 0.1 | 37.9 + 16.8 | 0.24 + 0.13 | 8.5 + 3.6 | 12.7 + 8.5 | 24.5 + 15.7 |
| 141 RH | 4.1 + 0.1 | 44.0 + 11.0 | 0.72 + 0.19 | 20.0 + 2.9 | 41.2 + 9.7 | 34.5 + 10.1 |
| 165 LH | 4.1 + 0.1 | 37.1 + 9.3 | 0.13 + 0.03 | 11.5 + 4.0 | 28.3 + 24.9 | 24.5 + 12.9 |
| **Mean + SD:** | **3.3 + 0.5** | **37.1 + 11.7** | **0.40 + 0.23** | **17.7 + 7.7** | **21.4 + 13.8** | **24.7 + 10.9** |

PEL length, KA width, KA displacement, and KA-SDL measurements derived from microscopy images as described in the Supplemental Methods (Additional file 11). PEL length is the entire length of the PEL from the base of the adjacent abaxial-most SELs to the tip of the axial-most SEL. KA width is measured at the approximate mid-way point along the same PELs used for PEL length. Displacement between the axial-most SEL and the KA (KA Disp) is also measured on the same PELs used for PEL length. KA-SDL distance is measured at three different locations along the PEL relative to the central axis of the foot: abaxial (adjacent to the hoof wall/stratum medium), middle, and axial (adjacent to the distal phalanx).

**ID**: Identification of individual feet evaluated; **Control**: Non-laminitic or mildly/subclinically affected (control) front feet; **EL Front**: Moderately to severely affected front feet from horses with endocrinopathic laminitis; **EL Hind**: Non-laminitic or mildly/subclinically affected hind feet from horses with endocrinopathic laminitis; **KA:** Keratinized Axis of primary epidermal lamella; **NA:** Not Analyzed (lamellar anatomy too disrupted to permit identification and measurement of the anatomical features); **PEL:** Primary Epidermal Lamella; **SDL:** Secondary Dermal Lamella; **LF:** Left Front foot; **LH:** Left Hind foot; **RF:** Right Front foot; **RH:** Right Hind foot.

Each value listed for individual feet represents the mean and standard deviations (SD) of five different measurements. The overall mean and SD for each group are shown below the data for individual feet. Since data were not normally distributed, mean measurements were compared between groups using Kruskal-Wallis One Way Analysis of Variance (ANOVA) on Ranks followed by all pairwise multiple comparison using Dunn’s Method.

**Differs from EL Hind and Control (P<0.05).
