# Supplementary material for: Detection of endoplasmic reticulum stress and the unfolded protein response in naturally-occurring endocrinopathic equine laminitis
Source: BMC Vet Res. 2019 Jan 10;15:24. doi: 10.1186/s12917-018-1748-x (PMC6327420; doi:10.1186/s12917-018-1748-x)
Supplement: Supplementary file 3 — Table S3. Quantitative Histology Measurements II: SEL length, width and angle. This table summarizes measurements of the mean length, width and angle of secondary epidermal lamellae (SEL) of mid-dorsal lamellae from the front and hind feet of horses with Endocrinopathy-Associated Laminitis (EL) and front feet from control horses. (DOCX 23 kb) [file 12917_2018_1748_MOESM3_ESM.docx]

| **Table A3: Quantitative Histology Measurements II: SEL length, width and angle.** | | | | | | | |
| --- | --- | --- | --- | --- | --- | --- | --- |
| **ID** | **SEL Length (μm)** | | | **SEL Width (μm)** | | | **SEL-PEL Angle (degrees)** |
| **Control** | **Abaxial** | **Middle** | **Axial** | **Abaxial** | **Middle** | **Axial** | **Middle** |
| 61 RF | 161.2 + 35.5 | 145.3 + 25.9 | 138.9 + 33.5 | 17.3 + 2.1 | 41.4 + 13.9 | 70.3 + 16.5 | 54.1 + 13.2 |
| 92 LF | 236.9 + 172.7 | 150.0 + 52.0 | 88.2 + 32.3 | 18.8 + 7.5 | 43.0 + 16.7 | 40.7 + 9.7 | 51.6 + 17.2 |
| 102 LF | 101.0 + 11.8 | 153.4 + 39.2 | 166.1 + 81.0 | 21.9 + 5.7 | 20.9 + 3.8 | 30.2 + 8.7 | 37.7 + 4.1 |
| 110 LF | 141.6 + 9.6 | 101.8 + 10.8 | 89.4 + 12.0 | 26.2 + 1.7 | 42.6 + 6.3 | 42.3 + 10.6 | 58.6 + 11.6 |
| 111 LF | 176.7 + 37.2 | 148.3 + 33.3 | 144.6 + 34.5 | 17.8 + 3.9 | 19.3 + 5.1 | 30.3 + 5.3 | 38.1 + 12.9 |
| 113 LF | 121.4 + 39.0 | 84.6 + 27.2 | 52.8 + 15.1 | 17.4 + 4.4 | 23.6 + 5.5 | 27.8 + 4.5 | 39.8 + 10.4 |
| 114 LF | 99.6 + 28.8 | 127.0 + 45.8 | 152.8 + 39.7 | 25.0 + 8.7 | 23.2 + 5.8 | 34.2 + 6.6 | 38.4 + 9.4 |
| 129 RF | 203.2 + 17.4 | 208.6 + 17.3 | 99.6 + 32.2 | 19.6 + 1.1 | 20.2 + 3.0 | 34.6 + 8.6 | 28.0 + 1.4 |
| **Mean + SD:** | **155.2 + 76.0** | **139.9 + 47.0** | **116.6 + 52.2** | **20.5 + 5.7** | **29.3 + 13.1** | **38.8 + 15.6** | **43.3 + 14.0** |
| **EL Front** |  |  |  |  |  |  |  |
| 63 RF | 198.0 + 75.3 | 160.0 + 29.1 | 151.8 + 66.5 | 38.2 + 16.3 | 34.5 + 7.6 | 38.6 + 11.0 | 48.5 + 15.2 |
| 63 LF | 188.8 + 36.9 | 191.4 + 42.2 | 244.8 + 30.5 | 19.4 + 3.2 | 24.4 + 3.9 | 37.7 + 10.9 | 29.9 + 9.1 |
| 73 LF | 260.4 + 16.6 | 210.7 + 18.3 | 127.0 + 16.4 | 25.1 + 2.0 | 24.6 + 3.4 | 45.4 + 10.7 | 24.6 + 1.3 |
| 75 RF | NA | NA | NA | NA | NA | NA | NA |
| 75 LF | 408.6 + 142.9 | 474.4 + 283.5 | 145.4 + 27.4 | 38.0 + 15.1 | 43.4 + 12.2 | 48.3 + 8.6 | 33.7 + 4.7 |
| 90 LF | 117.5 + 27.9 | 53.5 + 8.5 | 25.4 + 6.3 | 53.6 + 9.4 | 39.5 + 7.0 | NA | 69.1 + 21.5 |
| 101 RF | 415.0 + 79.2 | 447.8 + 126.2 | 297.1 + 257.2 | 15.4 + 9.9 | 21.0 + 3.8 | 46.9 + 27.4 | 11.5 + 3.6 |
| 104 RF | 170.2 + 48.1 | 455.2 + 76.4 | 249.7 + 39.9 | 23.5 + 4.6 | 31.9 + 4.3 | 31.9 + 4.9 | 14.0 + 2.7 |
| 109 LF | 116.1 + 17.9 | 101.5 + 11.7 | 191.0 + 80.2 | 47.6 + 8.3 | 45.0 + 4.1 | 42.2 + 7.2 | 44.5 + 33.1 |
| 116 LF | NA | NA | 151.7 + 99.4 | NA | NA | 21.3 + 14.1 | 71.7 + 46.7 |
| 116 RF | 772.5 + 431.6 | 605.5 + 160.0 | 818.5 + 409.1 | 29.3 + 6.3 | 26.0 + 3.6 | 28.2 + 4.8 | 14.4 + 5.3 |
| 134 RF | 109.8 + 23.4 | 387.3 + 198.5 | 285.5 + 135.1 | 25.0 + 5.1 | 19.4 + 4.5 | 39.0 + 25.0 | 31.1 + 25.0 |
| 134 LF | 206.9 + 50.8 | 79.4 + 63.7 | 234.6 + 50.0 | 11.9 + 1.9 | 39.1 + 12.1 | 121.3 + 56.8 | 125.1 + 39.1 |
| 140 LF | 316.0 + 50.0 | 403.4 + 154.9 | 186.6 + 60.6 | 24.0 + 2.3 | 32.5 + 11.8 | 44.9 + 18.5 | 19.9 + 9.4 |
| 141 LF | 125.4 + 8.9 | 340.1 + 128.3 | 400.5 + 115.2 | 14.4 + 4.8 | 13.7 + 5.4 | 16.8 + 4.3 | 14.9 + 5.6 |
| 141 RF | 122.2 + 22.8 | 107.9 + 38.4 | 364.0 + 71.4 | 32.3 + 19.8 | 22.8 + 4.3 | 13.4 + 3.8 | 26.0 + 4.6 |
| 165 LF | 85.5 + 24.7 | 186.5 + 76.1 | 188.2 + 71.3 | 31.6 + 8.2 | 33.0 + 15.8 | 63.1 + 26.9 | 31.7 + 13.6 |
| **Mean + SD:** | **240.9 + 208.2** | **280.3 + 201.2** | **253.9 + 212.6*** | **28.6 + 14.4** | **30.1 + 11.5** | **42.6 + 30.9** | **38.2 + 34.1** |
| **EL Hind** |  |  |  |  |  |  |  |
| 63 LH | 104.6 + 14.8 | 198.4 + 33.2 | 160.4 + 83.0 | 26.3 + 6.1 | 31.2 + 4.0 | 19.7 + 2.8 | 34.4 + 6.6 |
| 73 LH | 145.9 + 12.6 | 131.8 + 6.5 | 102.6 + 8.0 | 26.3 + 5.2 | 30.8 + 5.0 | 44.5 + 6.6 | 45.2 + 5.8 |
| 75 RH | 99.2 + 29.6 | 117.8 + 33.0 | 102.2 + 32.2 | 23.9 + 8.0 | 30.2 + 7.7 | 31.2 + 13.2 | 48.3 + 25.3 |
| 101 LH | 181.0 + 45.3 | 164.2 + 15.8 | 130.8 + 33.6 | 21.9 + 4.0 | 21.1 + 3.7 | 22.6 + 7.1 | 45.9 + 6.7 |
| 104 RH | 192.4 + 82.1 | 342.6 + 103.6 | 214.2 + 87.2 | 20.1 + 1.7 | 24.9 + 15.9 | 28.6 + 7.5 | 28.0 + 3.9 |
| 109 RH | 99.8 + 17.5 | 175.8 + 17.0 | 184.3 + 14.5 | 31.7 + 4.6 | 30.9 + 3.4 | 34.9 + 8.5 | 42.0 + 11.7 |
| 116 RH | 84.8 + 16.4 | 113.0 + 16.4 | 98.6 + 9.0 | 31.0 + 4.9 | 35.0 + 3.3 | 31.8 + 1.3 | 49.2 + 7.4 |
| 134 RH | 129.6 + 49.4 | 94.4 + 20.0 | 116.0 + 31.6 | 19.2 + 4.3 | 26.4 + 6.5 | 32.8 + 15.3 | 53.9 + 7.5 |
| 141 RH | 112.7 + 18.5 | 202.6 + 71.1 | 172.2 + 19.0 | 34.0 + 5.4 | 41.9 + 9.3 | 47.1 + 12.8 | 43.2 + 13.0 |
| 165 LH | 175.7 + 21.4 | 230.2 + 80.0 | 140.5 + 28.7 | 23.7 + 3.1 | 26.3 + 5.3 | 54.3 + 15.6 | 34.0 + 4.0 |
| **Mean + SD:** | **132.6 + 50.2** | **177.1 + 83.6** | **142.2 + 54.8** | **25.8 + 6.6** | **29.9 + 8.7** | **34.8 + 13.9** | **42.4 + 12.9** |

SEL length, SEL width, and SEL-PEL angle measurements derived from microscopy images as described in the Supplemental Methods (Additional file 11). SEL length and SEL width measured at three different locations along the PEL relative to the central axis of the foot: abaxial (adjacent to the hoof wall/stratum medium), middle, and axial (adjacent to the distal phalanx). SEL-PEL angle measured at middle PEL location with base of angle along edge of KA, hypotenuse along axis of SEL, and point of angle oriented abaxially.

**ID**: Identification of individual feet evaluated; **Control**: Non-laminitic or mildly/subclinically affected (control) front feet; **EL Front**: Moderately to severely affected front feet from horses with endocrinopathic laminitis; **EL Hind**: Non-laminitic or mildly/subclinically affected hind feet from horses with endocrinopathic laminitis; **NA:** Not Analyzed (lamellar anatomy too disrupted to permit identification and measurement of the anatomical features); **PEL:** Primary Epidermal Lamella; **SEL:** Secondary Epidermal Lamella; **LF:** Left Front foot; **LH:** Left Hind foot; **RF:** Right Front foot; **RH:** Right Hind foot.

Each value listed for individual feet represents the mean and standard deviations (SD) of five different measurements. The overall mean and SD for each group are shown below the data for individual feet. Since data were not normally distributed, mean measurements were compared between groups using Kruskal-Wallis One Way Analysis of Variance (ANOVA) on Ranks followed by all pairwise multiple comparison using Dunn’s Method.

*Differs from Control, but not from EL Hind (P<0.05).
