# Supplementary material for: Detection of endoplasmic reticulum stress and the unfolded protein response in naturally-occurring endocrinopathic equine laminitis
Source: BMC Vet Res. 2019 Jan 10;15:24. doi: 10.1186/s12917-018-1748-x (PMC6327420; doi:10.1186/s12917-018-1748-x)
Supplement: Supplementary file 4 — Table S4. Overall qualitative histopathology severity score and histopathology lesion distribution scores. This table summarizes qualitative histopathology scores from the evaluation of H&E and PASH stained lamellar tissue sections by light microscopy, as described in the Supplemental Methods section. The overall (additive) histopathology score is listed for each limb and is derived from distribution scores for specific types of lesions that are also listed on this table (Lamellar Epidermal Basal Cell Pleomorphism, Basement Membrane, KA-axial SEL displacement, SEL Morphology, Epidermal Pathology, Dermal Spindle Cell Hypertrophy, Dermal Interstitial Pathology, White Blood Cells, and Vascular Pathology) and Table A5 (Lamellar Apoptotic/Necrotic and Mitotic Figure distribution scores). (DOCX 23 kb) [file 12917_2018_1748_MOESM4_ESM.docx]

| **Table A4: Overall qualitative histopathology severity score and histopathology lesion distribution scores.** | | | | | | | | | | | |
| --- | --- | --- | --- | --- | --- | --- | --- | --- | --- | --- | --- |
| **ID** | **Overall**  **Score** | **LEBC PM** | **BM** | **KA-SEL Disp** | **SEL Morph** | **Epid Path** | **Epid Vac** | **Derm Hyp** | **Derm Inter** | **WBC** | **Vasc** |
| **Control** |  |  |  |  |  |  |  |  |  |  |  |
| 61 RF | 32 | 3 | 3 | 1 | 3 | 3 | 3 | 3 | 2 | 2 | 2 |
| 92 LF | 33 | 3 | 2 | 0 | 3 | 3 | 3 | 3 | 3 | 2 | 2 |
| 102 LF | 20 | 1 | 2 | 1 | 2 | 2 | 2 | 1 | 2 | 1 | 1 |
| 110 LF | 27 | 2 | 2 | 1 | 2 | 3 | 3 | 1 | 2 | 2 | 2 |
| 111 LF | 28 | 2 | 2 | 1 | 2 | 2 | 2 | 2 | 2 | 2 | 2 |
| 113 LF | 19 | 1 | 2 | 1 | 2 | 2 | 1 | 1 | 3 | 1 | 2 |
| 114 LF | 22 | 1 | 3 | 1 | 1 | 2 | 2 | 2 | 2 | 2 | 2 |
| 129 RF | 23 | 1 | 2 | 2 | 2 | 2 | 1 | 2 | 3 | 3 | 2 |
| **Mean + SD:** | **25.5 + 5.3** | **1.8 + 0.9** | **2.3 + 0.5** | **1.0 + 0.5** | **2.1 + 0.6** | **2.4 + 0.5** | **2.1 + 0.8** | **1.9 + 0.8** | **2.4 + 0.5** | **1.9 + 0.6** | **1.9 + 0.4** |
| **EL Front** |  |  |  |  |  |  |  |  |  |  |  |
| 63 RF | 41 | 4 | 3 | 4 | 3 | 4 | 3 | 4 | 3 | 2 | 3 |
| 63 LF | 37 | 3 | 3 | 3 | 3 | 3 | 3 | 3 | 3 | 2 | 2 |
| 73 LF | 33 | 3 | 2 | 1 | 3 | 3 | 3 | 3 | 2 | 4 | 3 |
| 75 RF | 45 | 4 | 4 | 4 | 4 | 4 | 0 | 4 | 4 | 4 | 4 |
| 75 LF | 47 | 4 | 4 | 4 | 4 | 4 | 2 | 4 | 3 | 2 | 4 |
| 90 LF | 34 | 3 | 3 | 4 | 3 | 3 | 2 | 2 | 2 | 4 | 2 |
| 101 RF | 48 | 4 | 4 | 4 | 4 | 4 | 2 | 4 | 3 | 3 | 3 |
| 104 RF | 44 | 4 | 3 | 3 | 4 | 4 | 3 | 3 | 3 | 3 | 3 |
| 109 LF | 43 | 3 | 3 | 3 | 3 | 4 | 3 | 3 | 3 | 3 | 3 |
| 116 LF | 43 | 4 | 4 | 4 | 4 | 4 | 2 | 4 | 2 | 3 | 3 |
| 116 RF | 46 | 4 | 4 | 4 | 4 | 4 | 2 | 4 | 3 | 3 | 3 |
| 134 RF | 44 | 4 | 4 | 2 | 3 | 4 | 3 | 4 | 4 | 3 | 4 |
| 134 LF | 39 | 4 | 4 | 3 | 4 | 4 | 2 | 4 | 2 | 3 | 3 |
| 140 LF | 37 | 3 | 3 | 4 | 4 | 4 | 3 | 3 | 3 | 3 | 2 |
| 141 LF | 41 | 4 | 4 | 4 | 4 | 4 | 3 | 3 | 3 | 2 | 3 |
| 141 RF | 45 | 4 | 4 | 4 | 4 | 4 | 2 | 4 | 3 | 3 | 3 |
| 165 LF | 38 | 3 | 3 | 3 | 3 | 3 | 3 | 3 | 3 | 3 | 3 |
| **Mean + SD:** | **41.5 + 4.5**** | **3.6 + 0.5**** | **3.5 + 0.6*** | **3.4 + 0.9**** | **3.6 + 0.5**** | **3.8 + 0.4**** | **2.4 + 0.8** | **3.5 + 0.6**** | **2.9 + 0.6** | **2.9 + 0.7**** | **3 + 0.6**** |
| **EL Hind** |  |  |  |  |  |  |  |  |  |  |  |
| 63 LH | 33 | 3 | 3 | 0 | 2 | 3 | 3 | 2 | 3 | 2 | 2 |
| 73 LH | 24 | 2 | 2 | 1 | 2 | 2 | 2 | 2 | 2 | 2 | 2 |
| 75 RH | 29 | 2 | 2 | 1 | 3 | 3 | 3 | 3 | 3 | 2 | 2 |
| 101 LH | 36 | 3 | 3 | 0 | 3 | 4 | 4 | 3 | 2 | 2 | 3 |
| 104 RH | 30 | 3 | 3 | 1 | 3 | 2 | 3 | 2 | 3 | 2 | 2 |
| 109 RH | 23 | 1 | 2 | 0 | 2 | 2 | 2 | 3 | 2 | 2 | 2 |
| 116 RH | 30 | 2 | 3 | 1 | 3 | 3 | 2 | 3 | 3 | 3 | 3 |
| 134 RH | 26 | 2 | 3 | 0 | 2 | 2 | 2 | 1 | 2 | 1 | 3 |
| 141 RH | 24 | 3 | 3 | 1 | 2 | 2 | 2 | 1 | 2 | 2 | 1 |
| 165 LH | 27 | 2 | 3 | 1 | 3 | 2 | 2 | 2 | 1 | 2 | 2 |
| **Mean + SD:** | **28.2 + 4.2** | **2.3 + 0.7** | **2.7 + 0.5** | **0.6 + 0.5** | **2.5 + 0.5** | **2.5 + 0.7** | **2.5 + 0.7** | **2.2 + 0.8** | **2.3 + 0.7** | **2.0 + 0.5** | **2.2 + 0.6** |

**Overall Score:** The additive qualitative histopathology score based on the lesion distribution scores listed on this table and **Table S5**; **LEBC PM:** Lamellar Epidermal Basal Cell Pleomorphism; **BM:** Basement Membrane pathology (for specific lesions, see **Additional file 6**); **KA-SEL Disp:** Secondary Epidermal Lamellae (**SEL**) displaced or detached from the keratinized axis (**KA**) of the Primary Epidermal Lamella (**PEL)**; **SEL Morph:** Abnormal SEL Morphology (for specific lesions, see **Additional file 7**); **Epid Path:** Epidermal lesions that represent abnormal tissue differentiation (for specific lesions, see **Additional file 7**); **Epid Vac:** Lamellar epidermal basal cell cytoplasmic vacuolization; **Derm Hyp:** Dermal spindle cell hypertrophy; **Derm Inter:** Expanded Dermal Interstitial spaces or loss of dermal connective tissue; **WBC:** White Blood Cell/Inflammatory leukocyte distribution (for leukocyte types based on morphology, see **Additional file 8**); **Vasc:** Vascular lesions (for specific pathology lesions, see **Additional file 8**); **LF:** Left Front foot; **LH:** Left Hind foot; **RF:** Right Front foot; **RH:** Right Hind foot. Qualitative lesion distribution scoring described in detail in Supplemental Methods (File A9), distribution of lesions subjectively scored as (**1**) Focal; (**2**) Multifocal; (**3**) Regional; (**4**) Global.

**ID**: Identification of individual feet evaluated; **Control**: Non-laminitic or mildly/subclinically affected (control) front feet; **EL Front**: Moderately to severely affected front feet from horses with endocrinopathy and obesity-associated laminitis; **EL Hind**: Non-laminitic or mildly/subclinically affected hind feet from horses with endocrinopathy and obesity-associated laminitis; **LF:** Left Front foot; **LH:** Left Hind foot; **RF:** Right Front foot; **RH:** Right Hind foot.

The means and standard deviations (SD) for each lesion are shown below individual foot scores for the three groups. Since data were not normally distributed, mean measurements were compared between groups using Kruskal-Wallis One Way Analysis of Variance (ANOVA) on Ranks followed by all pairwise multiple comparison using Dunn’s Method.

*Differs from Control (P<0.05).

**Differs from EL Hind and Control (P<0.05).
