# Supplementary material for: Detection of endoplasmic reticulum stress and the unfolded protein response in naturally-occurring endocrinopathic equine laminitis
Source: BMC Vet Res. 2019 Jan 10;15:24. doi: 10.1186/s12917-018-1748-x (PMC6327420; doi:10.1186/s12917-018-1748-x)
Supplement: Supplementary file 5 — Table S5. Lamellar apoptotic/necrotic and mitotic figure distribution scores. Table summarizing apoptotic/necrotic cell and SEL mitotic figure distribution scores for samples used in the current study. (DOCX 20 kb) [file 12917_2018_1748_MOESM5_ESM.docx]

| **Table A5: Lamellar apoptotic/necrotic and mitotic figure distribution scores.** | | | | | | |
| --- | --- | --- | --- | --- | --- | --- |
| **ID** | **Apoptotic/Necrotic Epid Cell Score** | | | **SEL Mitotic Figure Score** | | |
| **Control** | **Abaxial** | **Middle** | **Axial** | **Abaxial** | **Middle** | **Axial** |
| 61 RF | 3 | 2 | 1 | 0 | 0 | 1 |
| 92 LF | 3 | 3 | 3 | 0 | 0 | 0 |
| 102 LF | 2 | 1 | 2 | 0 | 0 | 0 |
| 110 LF | 2 | 2 | 3 | 0 | 0 | 0 |
| 111 LF | 2 | 3 | 2 | 0 | 0 | 2 |
| 113 LF | 1 | 1 | 1 | 0 | 0 | 0 |
| 114 LF | 1 | 1 | 2 | 0 | 0 | 0 |
| 129 RF | 1 | 1 | 1 | 0 | 0 | 0 |
| **Mean + SD:** | **1.9 + 0.8** | **1.8 + 0.9** | **1.9 + 0.8** | **0** | **0** | **0.4 + 0.7** |
| **EL Front** |  |  |  |  |  |  |
| 63 RF | 3 | 2 | 1 | 1 | 0 | 1 |
| 63 LF | 3 | 2 | 2 | 0 | 1 | 1 |
| 73 LF | 3 | 2 | 2 | 0 | 0 | 1 |
| 75 RF | 4 | 4 | 1 | 0 | 0 | 0 |
| 75 LF | 4 | 1 | 1 | 0 | 2 | 2 |
| 90 LF | 3 | 3 | 1 | 0 | 1 | 0 |
| 101 RF | 4 | 2 | 2 | 0 | 2 | 2 |
| 104 RF | 3 | 4 | 3 | 0 | 0 | 1 |
| 109 LF | 4 | 4 | 3 | 0 | 0 | 1 |
| 116 LF | 4 | 1 | 1 | 0 | 1 | 2 |
| 116 RF | 4 | 2 | 1 | 0 | 2 | 2 |
| 134 RF | 3 | 2 | 2 | 0 | 0 | 2 |
| 134 LF | 2 | 2 | 1 | 0 | 0 | 1 |
| 140 LF | 2 | 1 | 1 | 1 | 0 | 0 |
| 141 LF | 2 | 2 | 1 | 0 | 1 | 1 |
| 141 RF | 3 | 2 | 1 | 0 | 2 | 2 |
| 165 LF | 2 | 2 | 2 | 0 | 0 | 2 |
| **Mean + SD:** | **3.1 + 0.8**** | **2.2 + 1.0** | **1.5 + 0.3** | **0.1 + 0.3** | **0.7 + 0.8** | **1.2 + 0.8*** |
| **EL Hind** |  |  |  |  |  |  |
| 63 LH | 3 | 2 | 1 | 1 | 2 | 1 |
| 73 LH | 3 | 1 | 1 | 0 | 0 | 0 |
| 75 RH | 2 | 1 | 2 | 0 | 0 | 0 |
| 101 LH | 2 | 2 | 2 | 0 | 1 | 2 |
| 104 RH | 2 | 2 | 2 | 0 | 0 | 0 |
| 109 RH | 2 | 2 | 1 | 0 | 0 | 0 |
| 116 RH | 2 | 1 | 1 | 0 | 0 | 0 |
| 134 RH | 2 | 2 | 3 | 1 | 0 | 0 |
| 141 RH | 1 | 2 | 2 | 0 | 0 | 0 |
| 165 LH | 2 | 1 | 3 | 0 | 0 | 1 |
| **Mean + SD:** | **2.1 + 0.6** | **1.6 + 0.5** | **1.8 + 0.8** | **0.2 + 0.4** | **0.3 + 0.7** | **0.4 + 0.7** |

Apoptotic/Necrotic Epidermal (Epid) Cell and SEL Mitotic Figure distribution scores derived from cell counts obtained via light microscopy, as described in the Supplemental Methods (Additional file 11), for three different anatomical locations along the PEL relative to the central axis of the foot: **abaxial** (adjacent to the hoof wall/stratum medium), **middle**, and **axial** (adjacent to the distal phalanx). Cells were counted in three 40x fields (hpf) for each anatomical location, averaged, and assigned the distribution scores reported in this table as follows: Apoptotic/Necrotic Epid Cell distribution scored as (**1**) Focal (<10 cells/hpf), (**2**) Multifocal (10-30/hpf), (**3**) Regional (31-99/hpf), or (**4**) Global (>100/hpf). Mitotic Figure distribution scored as (**0**) None, (**1**) Few (<1/hpf), or (**2**) Many (>1/hpf).

The Apoptotic/Necrotic Epid Cell and SEL Mitotic Figure distribution score mean and standard deviations (SD) for each anatomical location are shown below individual foot scores for the three groups.

**ID**: Identification of individual feet evaluated; **Control**: Non-laminitic or mildly/subclinically affected (control) front feet; **EL Front**: Moderately to severely affected front feet from horses with endocrinopathic laminitis; **EL Hind**: Non-laminitic or mildly/subclinically affected hind feet from horses with endocrinopathic laminitis; **SEL:** Secondary Epidermal Lamella; **LF:** Left Front foot; **LH:** Left Hind foot; **RF:** Right Front foot; **RH:** Right Hind foot.

**Differs from EL Hind and Control (P<0.05), Kruskal-Wallis One Way Analysis of Variance (ANOVA) on Ranks followed by all pairwise multiple comparison using Dunn’s Method.

*Differs from Control (P=0.017), Kruskal-Wallis One Way Analysis of Variance (ANOVA) on Ranks followed by Mann-Whitney Rank Sum Test.
