# Supplementary material for: Detection of endoplasmic reticulum stress and the unfolded protein response in naturally-occurring endocrinopathic equine laminitis
Source: BMC Vet Res. 2019 Jan 10;15:24. doi: 10.1186/s12917-018-1748-x (PMC6327420; doi:10.1186/s12917-018-1748-x)
Supplement: Supplementary file 6 — Table S6. Qualitative Lamellar Basement Membrane histopathology lesion distribution scores. Table summarizing individual basement membrane (BM) histopathological lesion distribution scores that contributed to the BM pathology score reported in Table A4 for samples used in the current study, including distributions of thickened BM, fragmented BM, BM detached at SEL tips, SDL retraction, and completely detached BM. (DOCX 19 kb) [file 12917_2018_1748_MOESM6_ESM.docx]

| **Table A6: Qualitative Lamellar Basement Membrane histopathology lesion distribution scores.** | | | | | |
| --- | --- | --- | --- | --- | --- |
| **ID** | **Thickened** | **Fragmented** | **Detached at SEL tips** | **SDL retraction** | **Completely Detached** |
| **Control** |  |  |  |  |  |
| 61 RF | 2 | 3 | 2 | 0 | 0 |
| 92 LF | 2 | 2 | 2 | 0 | 0 |
| 102 LF | 1 | 0 | 2 | 0 | 0 |
| 110 LF | 2 | 2 | 1 | 0 | 0 |
| 111 LF | 2 | 1 | 2 | 2 | 0 |
| 113 LF | 2 | 0 | 2 | 0 | 0 |
| 114 LF | 3 | 3 | 3 | 2 | 0 |
| 129 RF | 0 | 0 | 2 | 2 | 0 |
| **Mean + SD:** | **1.8 + 0.9** | **1.4 + 1.3** | **2.0 + 0.5** | **0.8 + 1.0** | **0** |
| **EL Front** |  |  |  |  |  |
| 63 RF | 3 | 3 | 2 | 4 | 0 |
| 63 LF | 3 | 2 | 2 | 4 | 0 |
| 73 LF | 2 | 2 | 2 | 2 | 0 |
| 75 RF | 4 | 4 | 0 | 4 | 0 |
| 75 LF | 4 | 4 | 4 | 2 | 0 |
| 90 LF | 3 | 2 | 3 | 3 | 0 |
| 101 RF | 3 | 4 | 2 | 4 | 0 |
| 104 RF | 2 | 3 | 2 | 3 | 0 |
| 109 LF | 3 | 3 | 2 | 3 | 0 |
| 116 LF | 4 | 4 | 3 | 4 | 3 |
| 116 RF | 3 | 4 | 3 | 4 | 0 |
| 134 RF | 3 | 4 | 3 | 4 | 0 |
| 134 LF | 3 | 4 | 2 | 4 | 0 |
| 140 LF | 3 | 3 | 2 | 3 | 0 |
| 141 LF | 4 | 4 | 0 | 4 | 0 |
| 141 RF | 3 | 4 | 3 | 4 | 3 |
| 165 LF | 2 | 3 | 1 | 3 | 0 |
| **Mean + SD:** | **3.1 + 0.7*** | **3.4 + 0.8**** | **2.1 + 1.1** | **3.5 + 0.7**** | **0.4 + 1.0** |
| **EL Hind** |  |  |  |  |  |
| 63 LH | 3 | 3 | 2 | 3 | 0 |
| 73 LH | 1 | 1 | 1 | 2 | 0 |
| 75 RH | 3 | 2 | 2 | 0 | 0 |
| 101 LH | 2 | 3 | 0 | 1 | 0 |
| 104 RH | 2 | 2 | 2 | 3 | 0 |
| 109 RH | 3 | 2 | 2 | 2 | 0 |
| 116 RH | 3 | 1 | 0 | 3 | 0 |
| 134 RH | 3 | 2 | 2 | 2 | 0 |
| 141 RH | 3 | 0 | 0 | 3 | 0 |
| 165 LH | 2 | 3 | 2 | 3 | 0 |
| **Mean + SD:** | **2.5 + 0.7** | **1.9 + 1.0** | **1.3 + 0.9** | **2.2 + 1.0** | **0** |

Individual basement membrane histological lesion distribution scores that contributed to the Basement Membrane (BM) Pathology score reported in Table A4, as described in Supplemental Methods (Additional file 11): Thickened BM, Fragmented BM, BM detached from SEL tips, SDL and associated BM retracted from keratinized axis of the primary epidermal lamella, or BM completely detached from SEL. Distribution of lesions subjectively scored as (**1**) Focal; (**2**) Multifocal; (**3**) Regional; (**4**) Global.

**ID**: Identification of individual feet evaluated; **Control**: Non-laminitic or mildly/subclinically affected (control) front feet; **EL Front**: Moderately to severely affected front feet from horses with endocrinopathic laminitis; **EL Hind**: Non-laminitic or mildly/subclinically affected hind feet from horses with endocrinopathic laminitis; **SDL:** Secondary Dermal Lamella; **SEL:** Secondary Epidermal Lamella; **LF:** Left Front foot; **LH:** Left Hind foot; **RF:** Right Front foot; **RH:** Right Hind foot.

The means and standard deviations (SD) for each lesion are shown below individual foot scores for the three groups. Since data were not normally distributed, mean measurements were compared between groups using Kruskal-Wallis One Way Analysis of Variance (ANOVA) on Ranks followed by all pairwise multiple comparison using Dunn’s Method.

*Differs from Control (P<0.05).

**Differs from EL Hind and Control (P<0.05).
