# Supplementary material for: Detection of endoplasmic reticulum stress and the unfolded protein response in naturally-occurring endocrinopathic equine laminitis
Source: BMC Vet Res. 2019 Jan 10;15:24. doi: 10.1186/s12917-018-1748-x (PMC6327420; doi:10.1186/s12917-018-1748-x)
Supplement: Supplementary file 7 — Table S7. Qualitative lamellar epidermal histopathology lesion distribution scores. Table summarizing individual SEL morphology and epidermal differentiation lesions that contributed to the SEL Morph and Epid Path distribution scores reported in Table A4. Individual SEL morphology lesions include axial PEL blunting, SEL islands, abnormal SEL shape, merged SELs, and necrotic SELs. Individual epidermal differentiation lesions include hyperplasia, metaplasia, and orthokeratosis. (DOCX 21 kb) [file 12917_2018_1748_MOESM7_ESM.docx]

| **Table A7: Qualitative lamellar epidermal histopathology lesion distribution scores.** | | | | | | | | |
| --- | --- | --- | --- | --- | --- | --- | --- | --- |
| **ID** | **SEL Morphology** | | | | | **Epidermal Pathology** | | |
| **Control** | **Axial Blunting** | **Islands** | **Shape** | **Merged** | **Necrotic** | **Hyper-plasia** | **Meta-plasia** | **Ortho-keratosis** |
| 61 RF | 0 | 1 | 2 | 2 | 0 | 3 | 1 | 0 |
| 92 LF | 3 | 2 | 3 | 0 | 2 | 2 | 0 | 2 |
| 102 LF | 1 | 0 | 2 | 0 | 0 | 2 | 0 | 2 |
| 110 LF | 0 | 0 | 2 | 0 | 0 | 2 | 0 | 0 |
| 111 LF | 2 | 0 | 2 | 2 | 0 | 2 | 0 | 0 |
| 113 LF | 2 | 0 | 2 | 0 | 0 | 2 | 0 | 1 |
| 114 LF | 1 | 1 | 1 | 0 | 0 | 2 | 0 | 2 |
| 129 RF | 2 | 2 | 3 | 0 | 0 | 3 | 2 | 2 |
| **Mean + SD:** | **1.4 + 1.1** | **0.8 + 0.9** | **1.9 + 0.8** | **0.5 + 0.9** | **0.3 + 0.7** | **2.3 + 0.5** | **0.4 + 0.7** | **1.1 + 1.0** |
| **EL Front** |  |  |  |  |  |  |  |  |
| 63 RF | 0 | 2 | 3 | 3 | 0 | 4 | 0 | 4 |
| 63 LF | 0 | 3 | 3 | 3 | 0 | 4 | 2 | 3 |
| 73 LF | 0 | 3 | 3 | 3 | 2 | 3 | 2 | 2 |
| 75 RF | 0 | 4 | 4 | 4 | 3 | 0 | 4 | 4 |
| 75 LF | 0 | 4 | 4 | 4 | 3 | 4 | 4 | 4 |
| 90 LF | 0 | 1 | 3 | 3 | 3 | 3 | 3 | 4 |
| 101 RF | 0 | 4 | 4 | 4 | 3 | 4 | 4 | 4 |
| 104 RF | 0 | 1 | 4 | 4 | 3 | 4 | 3 | 4 |
| 109 LF | 0 | 2 | 4 | 3 | 3 | 3 | 2 | 4 |
| 116 LF | 0 | 3 | 4 | 4 | 3 | 4 | 4 | 4 |
| 116 RF | 0 | 3 | 4 | 4 | 3 | 4 | 4 | 4 |
| 134 RF | 0 | 3 | 3 | 3 | 3 | 4 | 3 | 3 |
| 134 LF | 0 | 3 | 4 | 4 | 0 | 4 | 4 | 4 |
| 140 LF | 0 | 4 | 4 | 4 | 3 | 3 | 3 | 4 |
| 141 LF | 0 | 4 | 4 | 4 | 3 | 4 | 2 | 4 |
| 141 RF | 0 | 3 | 4 | 4 | 3 | 4 | 3 | 4 |
| 165 LF | 0 | 3 | 3 | 3 | 2 | 3 | 3 | 3 |
| **Mean + SD:** | **0*** | **2.9 + 1.0**** | **3.6 + 0.5**** | **3.6 + 0.5**** | **2.4 + 1.2**** | **3.5 + 1.0**** | **2.9 + 1.1**** | **3.7 + 0.6**** |
| **EL Hind** |  |  |  |  |  |  |  |  |
| 63 LH | 0 | 1 | 2 | 2 | 0 | 3 | 3 | 3 |
| 73 LH | 1 | 2 | 2 | 2 | 1 | 2 | 2 | 2 |
| 75 RH | 0 | 3 | 3 | 2 | 0 | 2 | 0 | 0 |
| 101 LH | 0 | 2 | 3 | 0 | 0 | 0 | 0 | 0 |
| 104 RH | 2 | 1 | 3 | 2 | 0 | 2 | 1 | 2 |
| 109 RH | 0 | 0 | 2 | 2 | 0 | 0 | 0 | 2 |
| 116 RH | 2 | 2 | 3 | 3 | 2 | 3 | 3 | 3 |
| 134 RH | 3 | 2 | 2 | 0 | 2 | 0 | 0 | 0 |
| 141 RH | 0 | 0 | 2 | 0 | 0 | 2 | 2 | 3 |
| 165 LH | 1 | 3 | 3 | 2 | 0 | 0 | 2 | 2 |
| **Mean + SD:** | **0.9 + 1.1** | **1.6 + 1.1** | **2.5 + 0.5** | **1.5 + 1.1** | **0.5 + 0.8** | **1.4 + 1.3** | **1.3 + 1.3** | **1.7 + 1.3** |

Individual SEL morphology and epidermal differentiation lesions that contributed to the SEL Morph and Epid Path distribution scores reported in Table A4, as described in Supplemental Methods (Additional file 11): **Axial Blunting**: Axial tips of PELs and axial SELs have a blunted/crushed appearance, **Islands**: Epidermal islands consisting of epithelium or individual keratinocytes that are detached from the SEL, **Shape**: Abnormal SEL shape, including short, long, branched, **Merged:** Adjacent SELs on the same PEL merge or SELs on opposing PELs merge, **Necrotic**: All or most of the SEL has necrotic cells, **Hyperplasia:** Increased number of suprabasal cell layers in SELs, **Metaplasia:** PAS+ epidermal cells, **Orthokeratosis:** Increased cornification along KA or within SELs, formation of caphorn tubules and lamellar wedge tissue. Distribution of pathological lesions subjectively scored as (**1**) Focal; (**2**) Multifocal; (**3**) Regional; (**4**) Global.

**ID**: Identification of individual feet evaluated; **Control**: Non-laminitic or mildly/subclinically affected (control) front feet; **EL Front**: Moderately to severely affected front feet from horses with endocrinopathic laminitis; **EL Hind**: Non-laminitic or mildly/subclinically affected hind feet from horses with endocrinopathic laminitis; **PEL:** Primary Epidermal Lamella; **SDL:** Secondary Dermal Lamella; **SEL:** Secondary Epidermal Lamella; **LF:** Left Front foot; **LH:** Left Hind foot; **RF:** Right Front foot; **RH:** Right Hind foot.

The means and standard deviations (SD) for each lesion are shown below individual foot scores for the three groups. Since data were not normally distributed, mean measurements were compared between groups using Kruskal-Wallis One Way Analysis of Variance (ANOVA) on Ranks followed by all pairwise multiple comparison using Dunn’s Method.

*Differs from Control (P<0.05).

**Differs from EL Hind and Control (P<0.05).
