# Supplementary material for: Detection of endoplasmic reticulum stress and the unfolded protein response in naturally-occurring endocrinopathic equine laminitis
Source: BMC Vet Res. 2019 Jan 10;15:24. doi: 10.1186/s12917-018-1748-x (PMC6327420; doi:10.1186/s12917-018-1748-x)
Supplement: Supplementary file 9 — Additional validation of immunoblot band intensity measurements and comparison of two commercial antibodies to Grp78/BiP. The figure shows two plots to provide additional validation of the immunoblot band intensity measurements. (DOCX 21 kb) [file 12917_2018_1748_MOESM9_ESM.docx]

| **Table A8: Qualitative lamellar leukocyte and vascular histopathology lesion distribution scores.** | | | | | | | | |
| --- | --- | --- | --- | --- | --- | --- | --- | --- |
| **ID** | **Leukocyte Type** | | | | **Vascular Lesions** | | | |
| **Control** | **Mono** | **Plasma** | **PMN** | **Hemo** | **TE** | **PV Inflamm** | **Endo Act** | **Vasodil** |
| 61 RF | 2 | 0 | 1 | 3 | 0 | 0 | 2 | 2 |
| 92 LF | 2 | 2 | 0 | 0 | 2 | 0 | 2 | 2 |
| 102 LF | 1 | 0 | 0 | 1 | 0 | 0 | 1 | 0 |
| 110 LF | 2 | 2 | 0 | 1 | 0 | 0 | 2 | 2 |
| 111 LF | 2 | 1 | 0 | 0 | 0 | 1 | 2 | 2 |
| 113 LF | 1 | 0 | 0 | 1 | 0 | 1 | 0 | 2 |
| 114 LF | 2 | 1 | 0 | 2 | 0 | 0 | 2 | 2 |
| 129 RF | 2 | 3 | 0 | 2 | 0 | 0 | 2 | 2 |
| **Mean + SD:** | **1.8 + 0.5** | **1.1 + 1.1** | **0.1 + 0.4** | **1.3 + 0.9** | **0.3 + 0.5** | **1.6 + 0.7** | **1.8 + 0.7** | **1.5 + 1.3** |
| **EL Front** |  |  |  |  |  |  |  |  |
| 63 RF | 2 | 0 | 2 | 1 | 3 | 2 | 3 | 3 |
| 63 LF | 2 | 1 | 1 | 3 | 2 | 2 | 2 | 2 |
| 73 LF | 2 | 0 | 1 | 2 | 0 | 3 | 3 | 0 |
| 75 RF | 4 | 4 | 4 | 2 | 0 | 4 | 4 | 0 |
| 75 LF | 4 | 4 | 2 | 3 | 0 | 4 | 4 | 0 |
| 90 LF | 2 | 2 | 2 | 1 | 1 | 2 | 0 | 2 |
| 101 RF | 4 | 4 | 2 | 2 | 2 | 4 | 3 | 0 |
| 104 RF | 3 | 2 | 2 | 3 | 3 | 3 | 3 | 3 |
| 109 LF | 3 | 2 | 1 | 2 | 3 | 3 | 3 | 3 |
| 116 LF | 3 | 2 | 3 | 3 | 0 | 3 | 4 | 0 |
| 116 RF | 3 | 2 | 2 | 3 | 0 | 3 | 4 | 0 |
| 134 RF | 3 | 3 | 2 | 3 | 3 | 3 | 4 | 3 |
| 134 LF | 3 | 4 | 1 | 3 | 3 | 3 | 3 | 3 |
| 140 LF | 3 | 3 | 0 | 3 | 0 | 2 | 2 | 2 |
| 141 LF | 2 | 0 | 2 | 2 | 2 | 2 | 4 | 2 |
| 141 RF | 3 | 3 | 0 | 2 | 3 | 3 | 3 | 3 |
| 165 LF | 3 | 3 | 3 | 3 | 0 | 3 | 4 | 3 |
| **Mean + SD:** | **2.9 + 0.7**** | **2.3 + 1.4*** | **1.8 + 1.0**** | **2.4 + 0.7**** | **1.5 + 1.4** | **2.9 + 0.7**** | **3.1 + 1.1**** | **1.7 + 1.4** |
| **EL Hind** |  |  |  |  |  |  |  |  |
| 63 LH | 2 | 0 | 0 | 2 | 0 | 2 | 2 | 3 |
| 73 LH | 2 | 1 | 1 | 2 | 2 | 2 | 2 | 2 |
| 75 RH | 2 | 0 | 0 | 0 | 1 | 2 | 3 | 3 |
| 101 LH | 2 | 0 | 0 | 0 | 0 | 2 | 3 | 0 |
| 104 RH | 2 | 0 | 1 | 2 | 3 | 2 | 2 | 0 |
| 109 RH | 2 | 0 | 0 | 1 | 1 | 2 | 2 | 0 |
| 116 RH | 2 | 2 | 0 | 3 | 0 | 2 | 3 | 3 |
| 134 RH | 1 | 0 | 0 | 1 | 3 | 0 | 0 | 3 |
| 141 RH | 2 | 0 | 0 | 1 | 0 | 1 | 1 | 0 |
| 165 LH | 2 | 1 | 0 | 0 | 0 | 2 | 3 | 1 |
| **Mean + SD:** | **1.9 + 0.3** | **0.4 + 0.7** | **0.2 + 0.4** | **1.2 + 1.0** | **1.0 + 1.2** | **1.7 + 0.7** | **2.1 + 1.0** | **1.5 + 1.4** |

Individual leukocyte type (identified by morphology and staining) and Vascular Lesions that contributed to the WBC and Vasc distribution scores reported in Table A4 (as described in the Supplemental Methods (File A9): **Mono**: Mononuclear cells (histiocytes and lymphocytes), **Plasma**: Plasma cells, **PMN**: Polymorphonuclear leukocytes, **Hemo:** Hemosiderophages, **TE**: Intravascular thromboemboli, **PV Inflamm:** Perivascular inflammatory cell infiltration and/or adherent/extravasating inflammatory cells, **Endo Act:** Endothelial Activation, **Vasodil:** Vasodilation. Distribution of pathological features subjectively scored as (**1**) Focal; (**2**) Multifocal; (**3**) Regional; (**4**) Global.

**ID**: Identification of individual feet evaluated; **Control**: Non-laminitic or mildly/subclinically affected (control) front feet; **EL Front**: Moderately to severely affected front feet from horses with endocrinopathic laminitis; **EL Hind**: Non-laminitic or mildly/subclinically affected hind feet from horses with endocrinopathic laminitis; **PEL:** Primary Epidermal Lamella; **SDL:** Secondary Dermal Lamella; **SEL:** Secondary Epidermal Lamella; **LF:** Left Front foot; **LH:** Left Hind foot; **RF:** Right Front foot; **RH:** Right Hind foot.

The means and standard deviations (SD) for each histopathological feature are shown below individual foot scores for the three groups. Since data were not normally distributed, mean measurements were compared between groups using Kruskal-Wallis One Way Analysis of Variance (ANOVA) on Ranks followed by all pairwise multiple comparison using Dunn’s Method.

*Differs from EL Hind (P<0.05).

**Differs from EL Hind and Control (P<0.05).
