# Supplementary material for: Detection of endoplasmic reticulum stress and the unfolded protein response in naturally-occurring endocrinopathic equine laminitis
Source: BMC Vet Res. 2019 Jan 10;15:24. doi: 10.1186/s12917-018-1748-x (PMC6327420; doi:10.1186/s12917-018-1748-x)
Supplement: Supplementary file 10 — Additional validation of immunoblot band intensity measurements and comparison of two commercial antibodies to Grp78/BiP. Figure legend for additional file 9. (DOCX 23 kb) [file 12917_2018_1748_MOESM10_ESM.docx]

**Supplemental Methods**

Gross Pathology Scoring: Qualitative lesions compatible with laminitis were scored on sagittal sections and images of sagittal sections taken at the time of sectioning as follows: 1: No/minimal gross changes and stratum internum – corium measurement (SICM, measured in mm from the inner limit of the hoof wall/stratum medium to the dorsal surface of the distal phalanx at proximal, middle and distal locations [4]) within 2 sd from the mean middle SICM of the control light breed horses used in this study (5.7 + 1.0 mm, Table A1), 2: Some increase in SICM or rotation of the distal phalanx relative to the hoof capsule (>2 sd from control mean, < 2 mm difference between distal and proximal SICM) and/or hyperemic lamellar and sub-lamellar dermal tissue as evidence of hemorrhage, 3: Rotation (> 2 mm difference between distal and proximal SICM), hemorrhage, and/or lamellar wedge [8], 4: Any of the following: partially or fully detached hoof capsule, sinking, lamellar wedge, distal phalanx remodeling [4], crushed solar, coronary, or proximal parietal dermis, solar herniation. SICM measurements and gross pathology score for each limb are reported in Table A1 (see Additional File 1).

Quantitative Histology Measurements: Formalin-fixed/paraffin-embedded mid-dorsal lamellar tissue samples, including approximately 1 mm of hoof wall/stratum medium, the lamellae/stratum internum, and dermal tissue up to the dorsal surface of the distal phalanx, were used for histology studies of each foot. Separate 6 μm sections were stained with hematoxylin and eosin (H&E) stain and with Periodic acid-Schiff-hematoxylin (PASH) stain for each limb. Light microscopy images (Leica model DM5000B; Leica Biosystems: Buffalo Grove, IL) were captured with an Infinity 1-2CB camera (Lumenera: Ottawa, ON) using 1.25x, 10x, and 20x objectives and Image-Pro Plus (v. 7.0) image processing software (Media Cybernetics: Rockville, MD). The tiling feature for image capture was used for samples with elongated PELs or SELs that did not fit in the field of view. Scale bars for each objective were calibrated relative to a stage micrometer. “Abaxial,” “Middle,” and “Axial” anatomical locations refer to the axis of the digit, such that abaxial is adjacent to the hoof wall and axial is closest to the distal phalanx.

Five measurements were performed for each microanatomical feature. For each microanatomical feature assessed, the PEL or SEL to be measured was chosen by rolling a 6-sided die and counting from the edge of the section or the last chosen PEL or SEL in order to randomize selection. ImageJ (NIH: Bethesda, MD) image analysis software was used to take linear measurements that were calibrated to the distance in pixels, determined from the scale bar, for each image.

PEL length was measured along the keratinized axis (KA) using the image captured using the 1.25x objective and H&E stained slide and extended from the most abaxial end of of the KA (aligned with the bases of the most abaxial SELs to either side of the KA). KA width was measured on the same 1.25x image at the mid-way point along the same PELs as those used for PEL length. KA displacement was measured on the same 1.25x image, using the same PELs as those used for PEL length, as the distance from the axial end of the axial-most SEL to the axial-most tip of the KA.

KA-SDL distance was measured at axial, middle, and abaxial locations along the PEL using the PASH-stained slide and images captured with the 20x objective. PASH stains the basement membrane zone purple and is used to identify the tip of the SDL. KA-SDL distance was measured from the basement membrane at the tip of the SDL to the edge of the KA, in alignment with the axis of the SDL.

SEL length and width (midway along SEL length) were measured at axial, middle, and abaxial locations along the PEL using the H&E-stained slide and images captured with the 10x objective. SEL-PEL angle was measured on the same image at the middle location along the PEL with the base aligned with the edge of the KA and the hypotenuse along the axis of the SEL and the point of the angle oriented abaxially.

Distribution of Qualitative Histological Lesions Compatible with Laminitis and Overall Histopathology Severity Scoring: Qualitative histopathology scoring was performed by light microscopy of H&E and PASH stained mid-dorsal lamellar tissue sections using a Leica model DM5000B light microscope. The overall qualitative histopathological summary score was generated by adding the distribution scores for specific histopathology lesions (Tables A4-A5 (see Additional Files 4,5)), where 0 = not present, 1 = normal or focal, 2 = multifocal, 3 = regional, and 4 = global. Since histopathology is quite variable for limbs from horses with chronic laminitis, and published EL histopathology studies have focused on epidermal morphology changes [7, 22], this distribution scoring system allowed us to provide an overall description of each sample and to score severity based on the presence and distribution of multiple histological lesions, but did not quantify these lesions, and is similar to our previously reported scoring system [4].

Details of the qualitative histopathological lesions were as follows: Pleomorphic lamellar epidermal basal cell nuclear morphology (LEBC PM) is defined as rounded and inconsistently sized and oriented nuclei relative to the SEL axis. Basement membrane (BM) lesions encompass specific types of basement membrane lesions that were also scored for presence and distribution (Table A6 see Additional File 6)): thickened, fragmented, displaced from the SEL tip forming a point in the basement membrane or an acellular gap, retraction of the SDL/basement membrane tip away from the edge of the KA, and complete detachment of the basement membrane from the entire SEL. Displacement or detachment of secondary epidermal lamellae from the keratinized axes of the primary epidermal lamellae (KA-SEL Disp) was scored as 1 (normal/artifact) if the SEL cell morphology was normal and was assessed as a lesion (scored 2-4) if KA detachment or displacement was associated with necrotic/apoptotic cells, metaplastic cells, or if SELs shifted axially relative to the KA with gaps, necrotic material, and serum lakes left abaxially. Abnormal SEL morphology (SEL Morph) includes specific types of lesions that were also scored for presence and distribution (Table A7 (see Additional File 7)): detached epidermal cells or groups of cells (“islands”), abnormal SEL shape (including elongated, short, curved, or branched SELs), merged SELs (adjacent SELs on the same PEL or bridging to the opposite PEL), and necrotic SELs. Vacuolization of epidermal basal cells (Epid Vac) was defined as the presence of cytoplasmic vacuoles. Epidermal cell lesions (Epid Path) included hyperplasia (increased number of suprabasal cell layers beyond the usual 1-2 layers), metaplasia (detected with PASH staining as PAS-staining epidermal cells), and orthokeratosis (increased cornification of epidermal tissues, as evidenced by widening of the KA and/or cornifying SEL suprabasal cells). Dermal spindle cell hypertrophy (Derm Hyp) was detected as enlarged dermal spindle cell nuclei and/or increased numbers of dermal spindle cells. Expanded dermal interstitial space or loss of dermal connective tissue (Derm Inter) was detected as gaps in dermal tissue, especially in SDLs, and expanded extracellular space around SELs, especially axially. Inflammatory cell perivascular and/or dermal infiltration (WBC) included the presence and distributions of specific leukocytic cell types, based on morphology and staining, as detailed on Table A8 (see Additional File 8): Mononuclear cells (histiocytes and lymphocytes), plasma cells, polymorphonuclear leukocytes, and hemosiderophages. Vascular lesions (Vasc) includes specific lesions that were also scored for presence and distribution, as detailed in Table A8 (see Additional File 8): Intravascular thromboemboli, perivascular inflammation (inflammatory cells around vessels and/or adhering to/extravasating from vessels), endothelial hypertrophy (enlarged, prominent endothelial cell nuclei that protrude into the intravascular lumen), and vasodilation.

Mitotic figure and apoptotic/necrotic cell counts were also converted to distribution scores and incorporated into the overall qualitative histopathology score. Mitotic figure distribution was scored as 0: none were counted in three high power (40x objective) fields (hpf), 1: average of 1 or less counted in three hpf (“few”/focal), 2: average greater than 1 counted in three hpf (“frequent”/multifocal). Apoptotic/Necrotic cell distribution were based on average count for three hpf with cells identified on H&E stained slides by condensed, dark-staining nuclei without discernable nucleoli, increased cytoplasmic eosinophilia, and shrunken appearance or very pale and expanded cytoplasm and nucleus with loss of intercellular bridges (gaps between it and adjacent cells), as previously described [70,71]. Apoptotic/Necrotic cell distribution was scored as 1: < 10 average/hpf (focal), 2: 10-30 average/hpf (multifocal), 3: 31-99 average/hpf (regional), or 4: > 100 average/hpf (global).
